# All‐Round Talent: Unique Zinc Guanidine Catalyst Performs Efficiently in Synthesis and Chemical Recycling of (Bio)Polyesters

**DOI:** 10.1002/advs.202511260

**Published:** 2025-10-24

**Authors:** Tabea Becker, Koki Takeuchi, Carolin Süßmuth, Frederico Boekhoff, Martin A. Schäfer, Shunsuke Kato, Alexander Hoffmann, Takashi Hayashi, Sonja Herres‐Pawlis

**Affiliations:** ^1^ Institute of Inorganic Chemistry RWTH Aachen University Landoltweg 1a 52074 Aachen Germany; ^2^ Bioeconomy Science Center (BioSC) Forschungszentrum Jülich GmbH 52425 Jülich Germany; ^3^ Department of Applied Chemistry Graduate School of Engineering Osaka University 2‐1 Yamadaoka, Suita Osaka 565–0871 Japan; ^4^ Institute of Physical Chemistry RWTH Aachen University Melatener Straße 20 52074 Aachen Germany

**Keywords:** circular economy, polylactide, recycling, ring‐opening polymerization, zinc

## Abstract

In this study, two new hybrid guanidine ligands are presented that serve as the basis for the preparation of six new zinc‐hybrid guanidine complexes. The complex [Zn{(*R*,*R*)TMGNMe_2_(1,2)ch}_2_](OTf)_2_ (**C1**) shows a very high catalytic activity toward lactide and caprolactone ring‐opening polymerization (ROP) under industrially relevant bulk conditions. Using recrystallized l‐lactide, polylactide with a molar mass of up to 118 000 g mol^−1^ can be produced. The extremely fast caprolactone polymerization highlights the versatility of **C1**, as the polymerization rate constants are of the same order of magnitude. This is further underlined by high activity toward the chemical recycling of polyesters. In particular, **C1** can be recycled highly efficiently, performing methanolysis and ethanolysis of polylactide up to nine times without any loss of activity. By combining efficient polymerization and depolymerization of (bio)polyesters, new catalyst paves the way toward a circular plastics economy.

## Graphical abstract

A new zinc hybrid guanidine catalyst [Zn{(*R*,*R*)TMGNMe_2_(1,2)ch}_2_](OTf)_2_ (**C1**) shows very high catalytic activity in the polymerization of cyclic esters and in the depolymerization of these polyesters. Using **C1** in the ring‐opening polymerization of lactide and caprolactone under industrially relevant conditions, astonishing rate constants of the same order of magnitude are obtained. The recyclability of the catalyst in methanolysis is particularly outstanding.

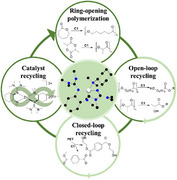

## 1 Introduction

Plastics have become indispensable in today's world, as these materials are used in almost every part of our daily lives due to their cost‐effective production and tailor‐made properties. Industrial, fossil‐based plastics production follows a linear economic model in which the materials end up in incinerators, landfills or the environment at their end‐of‐life (EoL), mostly after a single use.^[^ ^1^, ^2^ ^]^ The long‐term consequences of the accumulation of plastic waste in the environment pose a major challenge and lead to far‐reaching problems in ecosystems and the health of organisms.^[^ ^3^, ^4^, ^5^ ^]^ In 2023, global plastic production amounted to 413.8 Mt, with only <10% corresponding to a circular product.^[^ ^6^ ^]^ Due to limited resources, alternative ways of producing plastics are being sought. Therefore, circular economy schemes as well as bio‐based and bio‐attributed plastics production become key aspects of a sustainable and environmentally friendly plastics economy.^[^ ^7^, ^8^, ^9^ ^]^ Polylactide (PLA) is one of the most promising bio‐based, biodegradable bioplastics, already used in packaging materials and medical applications.^[^ ^10^, ^11^ ^]^ The monomer lactide (LA) is produced from corn, sugar cane or other sugar‐containing biomass (e.g., also from waste streams) via fermentation to lactic acid with subsequent dimerization. LA is then polymerized to the polyester PLA in a ring‐opening polymerization (ROP) via the metal‐catalyzed coordination‐insertion mechanism.^[^ ^12^, ^13^, ^14^, ^15^ ^]^ Another interesting biodegradable bioplastic is polycaprolactone (PCL), which is also obtained via the ROP of ε‐caprolactone (CL). Industrially, CL is synthesized using the Baeyer‐Villiger oxidation of fossil‐based cyclohexanone.^[^ ^16^ ^]^ However, recent studies show that CL can also be produced from a biobased resource utilizing C_6_ sugars with 5‐(hydroxymethyl)furfural and 1,6‐hexanediol.^[^ ^17^, ^18^ ^]^ Biodegradable bioplastics in combination with recycling methods incorporating a holistic view of the EoL scenario will lead to a reduction in environmental pollution with the goal of a circular economy.^[^ ^19^, ^20^ ^]^ Due to their reactive group in the polymer backbone, polyesters are particularly interesting for chemical recycling, which includes pyrolysis, hydrolysis, and alcoholysis. Alcoholysis recycles polymers by breaking their ester bonds via transesterification with different alcohols, resulting in a wide range of products. These can be utilized as platform chemicals, green solvents or monomers, creating value from EoL‐plastics.^[^ ^21^ ^]^ It can further be distinguished between open‐loop and closed‐loop schemes. For example, PLA can be valorized to the corresponding alkyl lactates in an open‐loop process, whereas in closed‐loop recycling of PET, the monomers dimethyl terephthalate (DMT) or bis(hydroxyethyl) terephthalate (BHET) can be recovered.^[^ ^22^, ^23^, ^24^, ^25^ ^]^

A more detailed examination of the ROP of PLA and PCL shows that the toxic catalyst tin(II) bis(2‐ethylhexanoate) (Sn(Oct)_2_) is used industrially with alcohol as a co‐initiator.^[^ ^26^, ^27^, ^28^, ^29^ ^]^ At low catalyst concentrations, colorless polymers with high molecular masses are obtained. In addition to food packaging, PLA and PCL are also used in medical applications, for example, for implants or as a controlled‐delivery drug preparation.^[^ ^30^, ^31^, ^32^, ^33^, ^34^ ^]^ The toxic properties of Sn(Oct)_2_ limit the widespread application of PLA, which fosters the intensive search for alternative, biocompatible metal catalysts. Meanwhile, the metals magnesium^[^ ^35^, ^36^, ^37^, ^38^ ^]^ iron^[^ ^39^, ^40^, ^41^, ^42^ ^]^ or zinc^[^ ^43^, ^44^, ^45^, ^46^, ^47^ ^]^ are widely reported in literature, but only in academic use. The biocompatible catalyst should show at least equivalent activity under industrially relevant conditions, which means that the metal complex has to be stable against impurities in the monomer and in the process.^[^ ^48^ ^]^ An industrially relevant catalyst is characterized by the fact that the ligand is commercially available and the catalyst is easy to synthesize in high yields. Furthermore, it should exhibit high activity in bulk polymerization, requiring the catalyst to be stable in the range of 130 to 180 °C. The polymerization should take place in high conversions with resulting polymers in high molar masses and narrow dispersities.^[^ ^48^ ^]^

In particular, zinc‐based catalysts are promising candidates for the polymerization of cyclic esters and chemical recycling due to their good biocompatibility. For example, Mazzeo et al. presented a heteroleptic pyridyl imino‐phenolate zinc complex, and Jones et al. ONS zinc complexes and zinc and magnesium complexes of tridentate ONN ligands that catalyze lactide polymerization and degradation under industrially relevant conditions.^[^ ^49^, ^50^, ^51^ ^]^ Besides the use of tailored zinc complexes as catalysts, Enthaler et al. demonstrated the polymerization and depolymerization of CL and PCL using microwave heating, respectively, with Zn(OAc)_2_ as a catalyst.^[^ ^52^ ^]^ Among these examples, zinc‐guanidine complexes are particularly interesting candidates, as these have proven to be highly active in the ROP of LA and CL, as well as tested as non‐hazardous in eco‐toxicological studies.^[^ ^53^, ^54^, ^55^, ^56^ ^]^ In 2020, Herres‐Pawlis and co‐workers presented a zinc triflate bisguanidine catalyst (*k*_p_  = 1.432 mol L^−1^ s^−1^, reaction time between 3 and 15 min) that is ten times more active in polymerization than the industrially used Sn(Oct)_2_ catalyst (*k*_p_  = 0.167 mol L^−1^ s^−1^).^[^ ^55^ ^]^ For the same catalyst also a high activity in the chemical recycling of (bio)polyesters was also demonstrated. This underlines the high potential of this catalyst type for use in a circular plastics economy.^[^ ^57^ ^]^

In this study, we present six new zinc hybrid guanidine complexes based on two new hybrid guanidine ligands, whereby the zinc triflate catalyst [Zn{(*R*,*R*)TMGNMe_2_(1,2)ch}_2_](OTf)_2_ (**C1**) demonstrates an enormously raised activity in the ROP of LA and – what is even more intriguing – a comparably high activity in both the ROP of LA and CL. To implement a circular plastics economy, the catalyst was also tested in the chemical recycling of polyesters and showed its very high efficiency. The recyclability of **C1** itself for the alcoholysis of PLA is particularly impressive here, with nine recycling cycles possible, making this catalyst an all‐round talent.

## 2 Results and Discussion

**Scheme 1:**
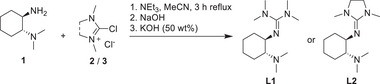
Ligand synthesis of TMGNMe_2_ch (L1) and DMEGNMe_2_ch (L2).

In recent years, our research group has been studying the design of zinc‐guanidine catalysts that are highly active in lactide polymerization. These catalysts have expanded their field of application toward the chemical recycling of polyesters, which illustrates the high potential of utilizing these catalyst systems in the circular plastics economy.^[^ ^58^, ^59^, ^60^ ^]^ *N*,*N*‐ligands with an aliphatic backbone are particularly active in contrast to their aromatic analogs. We have developed two new hybrid guanidine ligands that can be prepared in a one‐step synthesis following the procedure first described by Kantlehner et al. (**Scheme** **1**).^[^ ^61^ ^]^ The chiral amine **1** was reacted with two different Vilsmeier salts (TMG: tetramethylguanidine (**2**), DMEG: dimethylethyleneguanidine (**3**)), obtaining the new ligands TMGNMe_2_ch (**L1**) and DMEGNMe_2_ch (**L2**).

**Figure 1:**
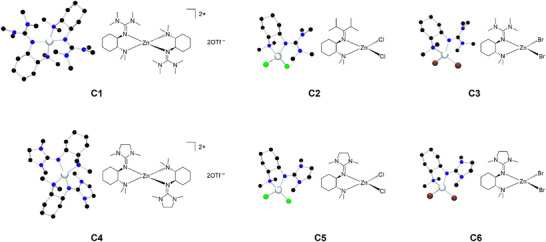
Molecular structures in the solid state of the complexes **C2**–**C3**, **C5**–**C6**, and the cationic complex units in **C1** and **C4**. H‐atoms and non‐coordinating anions are omitted for clarity.

Subsequently, both ligands were combined with three different zinc salts (zinc chloride, zinc bromide, and zinc triflate) and the six new zinc hybrid guanidine complexes (**C1**–**C6**) were obtained. The molecular structure in the solid state of the complexes was elucidated by single‐crystal X‐ray diffraction (XRD) analysis (**Figure** **1**, **Table** **1**). Two zinc triflate complexes [Zn{(*R*,*R*)TMGNMe_2_(1,2)ch}_2_](OTf)_2_ (**C1**) and [Zn{(*R*,*R*)DMEGNMe_2_(1,2)ch}_2_](OTf)_2_ (**C4**), two zinc chloride complexes [ZnCl_2_(*R*,*R*)TMGNMe_2_(1,2)ch] (**C2**) and [ZnCl_2_(*R*,*R*)DMEGNMe_2_(1,2)ch] (**C5**) as well as two zinc bromide complexes [ZnBr_2_(*R*,*R*)TMGNMe_2_(1,2)ch] (**C3**) and [ZnBr_2_(*R*,*R*)TMGNMe_2_(1,2)ch] (**C6**) were crystallized.

**Table 1:** Key geometric data of complexes **C1**–**C6**.

#	Zn–N_gua_ [Å]	Zn–N_Me2_ [Å]	N_gua_–Zn–N_Me2_ [°]	*ρ* ^a)^	*τ*_4_ ^b)^
**C1**	1.9939(15) 1.9829(16)	2.0775(16) 2.0930(15)	86.80(6) 86.40(6)^c)^	0.99 0.98	0.69
**C2**	2.0109(13)	2.1141(13)	84.42(5)	0.97	0.90
**C3**	2.0096(15)	2.1132(15)	84.53(6)	0.97	0.89
**C4**	1.975(5) 1.982(5)	2.070(5) 2.063(5)	87.4(2) 87.9(2)^c)^	0.98 0.97	0.74
**C5**	2.0174(10)	2.1078(10)	84.75(4)	0.96	0.90
**C6**	2.0187(15)	2.1012(15)	84.79(6)	0.96	0.90

^a)^ *ρ* = 2a/(b+c);^[^ ^62^ ^]^

^b)^ *τ*_4_ *=* [360°−(α+β)]/141°, with *τ*_4_ = 0 indicating square‐planar coordination and *τ*_4_ = 1 for tetrahedral coordination;^[^ ^63^ ^]^ and

^c)^ Two chelate angles of both ligands to the Zn atom are reported.

All complexes contain a delocalized guanidine unit, which can be described by the structure factor *ρ* (0.97–0.99, Table 1).^[^ ^62^ ^]^ The comparison of the structural parameter *τ*_4,_ which provides information about the coordination environment at the metal center is noteworthy.^[^ ^63^ ^]^ The zinc chloride and zinc bromide complexes show (as expected) an almost tetrahedral coordination (0.89–0.90, Table 1), whereas the zinc triflate complexes, in which two hybrid guanidine ligands coordinate to the zinc atom, reveal a distorted tetrahedral coordination motif (0.69 and 0.74, Table  1). A trend among all six complexes is visible comparing the Zn–N_gua_ bond length to Zn–NMe_2_ bond length. In general, all six complexes **C1**–**C6** show a shorter Zn–N_gua_ bond length, with the zinc triflate complexes **C1** and **C4** having the shortest bond lengths (Table 1). In order to verify the structural parameters, the literature known zinc‐*N*,*N*‐guanidine complexes [Zn(TMGhydroqu)_2_](OTf)_2_ and [Zn{(*R*,*R*)DMEG_2_(1,2)ch}_2_](OTf)_2_·THF with structural similarities were considered (Table S4, Supporting Information).^[^ ^54^, ^55^ ^]^ All complexes exhibit distorted‐tetrahedral coordination, whereby **C1** with a *τ*_4_ of 0.69 is located between the other two values of the literature complexes. It is noticeable that the Zn─N_gua_ bond lengths of the hybrid guanidine complexes are shorter compared to the bisguanidine complex. The shortest Zn─N_gua_ bond length is achieved with the hydroquinoline complex (1.959(3) Å and 1.950(3) Å). The Zn─N_amine_ bond length of **C1** is slightly longer compared to the hydroquinoline complex. There are also differences in the N─Zn─N bond angles. With 86.8(6) / 86.4(6), **C1** has a slightly larger N─Zn─N angle compared to the two zinc‐guanidine complexes known from literature.

Further analytical methods, such as nuclear magnetic resonance spectroscopy (NMR), infrared spectroscopy (IR), elemental analysis (CHN), and mass spectrometry (MS), also confirmed the structure of the complexes **C1**–**C6** and can be found in the supporting information.

### 2.1 Lactide and Caprolactone Polymerization Studies

To gain an insight into the activity of the presented catalysts **C1**–**C6**, polymerization experiments were carried out using recrystallized l‐lactide with a monomer‐to‐initiator‐ratio ([M]/[I]‐ratio) of 500:1 at 150 °C without solvent in Schlenk tubes (**Table** **2**; Figure S6, Supporting Information). Enormous differences in activity between the zinc chlorido and zinc bromido complexes compared to the zinc triflate complexes were observed. After 6 h of reaction time, the halide complexes (**C2**, **C3**, **C5,** and **C6**) only achieve a low lactide conversion of 9% to a maximum of 30% (Table 2). In sharp contrast to this stand the zinc triflate complexes (**C1** and **C4**) reach almost full conversion after only 3 min. Analysis of the ^1^H NMR spectrum of the crude polymerization mixture shows minimal *meso*‐lactide formation. To determine the tacticity of the polymer, homonuclear decoupled ^1^H NMR spectroscopy is used (Figure S11, Supporting Information). The spectrum shows isotactic PLA with a *P*_r_ of 0.14. The thermogravimetric analysis (TGA) measurements of complexes **C1** and **C4** show good thermal stability under industrially relevant conditions (at least 120 min at 150 and 200 °C, Figures S1 and S2, Supporting Information). The slightly increased dispersity of the polymers obtained with complexes **C1** and **C4** can be attributed to transesterification reactions. Significantly higher activity of these two complexes compared to the halide complexes is due to the non‐coordinating triflate anions, which results in a higher Lewis acidity, a trend is already known from the literature.^[^ ^54^, ^64^ ^]^

**Table 2:** Polymerization results of all complexes (**C1**–**C6**).

complex	*t* [min]^a)^	*X*_NMR_ ^b)^ [%]	*M*_n,exp_ ^c)^ [g mol^−1^]	*M*_n,theo_ ^d)^ [g mol^−1^]	*Ð* ^e)^
**C1**	3	92	83 400	66 300	1.6
**C2**	360	30	n.d.	21 600	n.d.
**C3**	360	25	n.d.	18 000	n.d.
**C4**	3	93	80 200	67 000	1.5
**C5**	360	9	n.d.	6 500	n.d.
**C6**	360	12	n.d.	8 600	n.d.

^a)^ Polymerization conditions: recrystallized l‐lactide (1.0 g, 0.007 mol), [M]/[I]‐ratio  =  500:1, *T*  =  150 °C, stirrer speed  =  260 rpm;

^b)^ Determined by ^1^H NMR spectroscopy;

^c)^ Determined via SEC analysis in THF;

^d)^ Calculated using the following equation: Molar mass × conversion × monomer ratio; and

^e)^ Calculated using the following equation: *M*_w_ / *M*_n_. n. d. = not determined.

**Figure 2:**
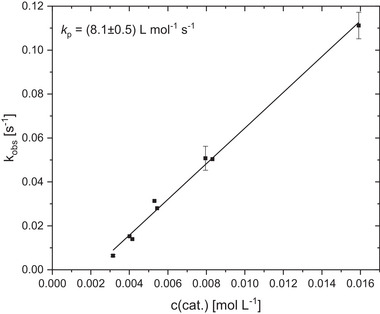
Plot of *k*_obs_ against catalyst concentration for the ROP of recrystallized l‐lactide catalyzed by C1 for the determination of the propagation rate constant *k*_p_. [M]/[I] = 500:1 to 2500:1. The polymerization straight line is not a straight line of origin due to a small extent of catalyst deactivation.

The most promising catalyst **C1** is easy to synthesize and was tested in detailed kinetic studies to determine its activity in the ROP of cyclic esters. Under solvent‐free conditions, lactide polymerization was carried out with variation of the catalyst concentration in a steel reactor at 150 °C under argon atmosphere (Figure S6, Supporting Information). The course of the reaction was monitored by in situ Raman spectroscopy. The resulting colorless polymers are important for industrial applications, as they enable plastic to be used in a wide range of applications. The observable reaction rate constant *k*_obs_ was determined for each catalyst concentration by a semilogarithmic plot of the monomer conversion against time (Figure S12, Supporting Information). The propagation rate constant *k*_p_ can be determined by plotting the obtained *k*_obs_‐value versus the catalyst concentration according to pseudo‐first‐order kinetics (**Figure** **2**). The linear regressions of the *k*_obs_ and *k*_p_ values indicate pseudo‐first‐order reaction kinetics, which are typical for the coordination‐insertion mechanism.^[^ ^43^, ^65^, ^66^, ^67^ ^]^

Table S6 (Supporting Information) shows all the important reaction parameters for lactide polymerization with variation of the catalyst concentration. High conversions and molar masses of up to 78 100 g mol^−1^ are achieved within a few seconds (75–165 s). As an example, polymerization at a [M]/[I] ratio of 1000:1 yields a high conversion of 94% within 75 s and a molar mass of the polymer of 57 300 g mol^−1^ and with a dispersity of 1.6 (Table S7, Supporting Information, entry 3). The experimentally determined molar masses are lower than the theoretically calculated values, and the dispersity of the polymers (1.4–1.9) are only slightly increased. Thermal decomposition of the catalyst has already been ruled out by TGA measurements, meaning that these results can mainly be attributed to transesterification reactions during polymerization. Furthermore, the viscosity of the reaction mixture increases rapidly within a short period of time, which inhibits mixing. Due to the mass transfer limitation at high viscosities and conversions, a conversion of 100% cannot be achieved. Removing the polymer from the reactor takes a few seconds, resulting in an extended heating period of the polymer, which might further promote the transesterification reactions.

**Figure 3:**
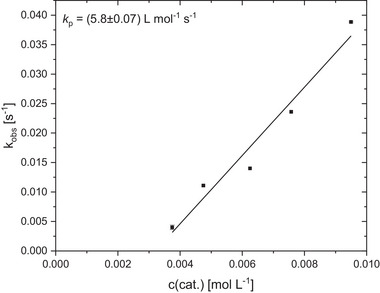
Plot of *k*_obs_ against catalyst concentration for the ROP of distilled ε‐caprolactone for the determination of the propagation rate constant *k*_p_. [M]/[I] = 1000:1 to 2500:1. The polymerization straight line is not a straight line of origin due to a small extent of catalyst deactivation.

To ensure better control of polymerization, experiments were carried out with the co‐initiator benzyl alcohol (BnOH) (Table S6, Supporting Information, entries 11 and 12). The molar mass approaches the theoretical value, but the dispersity of the obtained polymers is still increased. At a [M]/[BnOH]/[I] ratio of 1500:1:1, PLA with a molar mass of 118 000 g mol^−1^ was achieved, illustrating the extremely high activity of **C1**. Using BnOH as co‐initiator, higher conversions (up to 96%) were observed also with low catalyst loadings ([M]/[I] ratios of 2000:1 and 2500:1). A comparison with systems known from literature under the same reaction conditions is sometimes difficult, although this can help to contextualize the catalyst. Jones et al. presented the catalyst **CC1**, which achieves a conversion to PLA of 69% after only 1 min at a [M]:[I] ratio of 3000:1:10 and 130 °C (**Figure** **3**).^[^ ^50^ ^]^ In 2020, a very robust catalyst was presented by our research group. It is a zinc triflate bisguanidine complex [Zn{(*R*,*R*)‐DMEG_2_(1,2)ch}_2_](OTf)_2_·THF (**CC2**: *k*_p_ = 1.4323 mol L^−1^ s^−1^, techn. *rac*‐lactide, (Figure 3) with a similar structural motif. The comparison of the *k*_p_ values shows an ≈6‐fold increase in activity of the catalyst **C1** presented here, but it should be noted that the literature‐known catalyst was tested in polymerization with technical *rac*‐lactide.^[^ ^55^ ^]^

In order to fulfill industrial requirements, **C1** was additionally tested in polymerization with technical lactide ([M]/[I] ratio of 1000:1). No significant difference was observed in the polymerization results, which underlines **C1**’s industrial relevance (Table S7, Supporting Information, entry 4). The higher activity of **C1** in comparison to **CC2** might be explained by the better accessibility of the zinc center, which is only coordinated by two guanidine units and two amine donors (four guanidine units in the bisguanidine complex).

Solvent polymerization is particularly important for high‐performance materials in medical applications, making it necessary to test **C1** also in lactide ROP with an additional solvent (toluene). Similar to the above‐mentioned procedure the kinetic studies for the solvent polymerization with **C1** were carried out in Young‐type Schlenk tubes using recrystallized l‐lactide (1 g), toluene (8 mL), 100 °C, 260 rpm at varying catalyst concentrations ([M]/[I] ratios between 500:1 and 800:1). Note that there is no fixed reaction time because the stirring was conducted until the highest viscosity of the respective reaction mixture was reached to ensure the highest possible conversion. The catalyst **C1** is also highly active in the solvent polymerization of LA, and molar masses with good agreement to the theoretically calculated values are obtained (Table S7, Supporting Information). Compared to solvent‐free polymerization, polymers with increased dispersity (1.5–2.2) are also obtained here, underlining the ability of the catalyst for transesterification reactions. The well‐known zinc triflate hybrid guanidine catalyst ([Zn(TMGhydroqu)_2_](OTf)_2_) with a similar structural motif can be used as a comparative catalyst. In this case, experiments at corresponding [M]/[I] ratios with technical and recrystallized *rac*‐lactide in toluene yielded similar results. For **C1** a *k*_p_ value of 4.0 L mol^−1^ s^−1^ (Figure S14, Supporting Information) was determined. Compared to the catalyst from literature (*k*_p_ value: 1.1 L mol^−1^ s^−1^) this is 3.5 times higher.^[^ ^54^ ^]^ This further shows the high potential of **C1** as a versatile catalyst for lactide ROP. In 2023, Mazzeo et al. presented zinc‐based catalyst systems that enable rapid lactide polymerization in dichloromethane at room temperature (**CC3**: 100:1:1, 25 °C, 2 min, *X*_NMR_ = 84%, *M*_n,exp_ = 12 000 g mol^−1^, *Ð* = 1.12, (Figure 3).^[^ ^68^ ^]^

**Figure 4:**
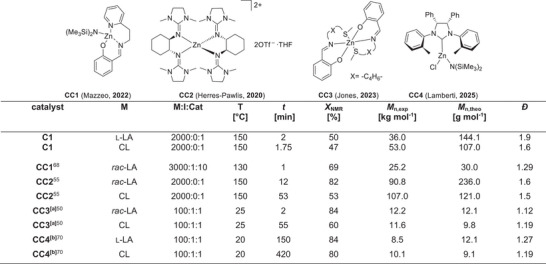
Comparison of **C1** with catalyst systems known from the literature. CC = Comparison catalyst. a) In CH_2_Cl_2_. b) In THF.

Due to the high activity in lactide polymerization, **C1** was also investigated toward the ROP of ε‐caprolactone (CL) under industrially relevant bulk conditions. As described for lactide ROP under these conditions, a kinetic study was performed, monitoring the polymerization by in situ Raman spectroscopy. Herein, distilled CL (8 mL) was polymerized at different catalyst concentrations ([M]/[I] ratios between 1000:1 to 2500:1) at 150 °C (**Figure** **4**; Table S9, Supporting Information).

As for lactide ROP **C1** shows a high activity in CL polymerization, yielding a *k*_p_ of 5.8 L mol^−1^ s^−1^. For the first time, a catalyst is presented with a catalytic activity in ε‐caprolactone polymerization, being in the same order of magnitude compared to the lactide polymerization. Previously reported guanidine‐based catalysts often show a difference in the rate constant of 30 up to two orders of magnitude between lactide and caprolactone ROP (**CC2**: *k*_p, PLA_  =  1.4323 L mol^−1^ s^−1^, *k*_p, PCL_  =  0.0487 L mol^−1^ s^−1^; [FeCl_2_(TMG5NMe_2_asme)]: *k*_p, PLA_  =  0.554 L mol^−1^ s^−1^, *k*_p, PCL_  =  0.00597 L mol^−1^ s^−1^).^[^ ^40^, ^55^, ^69^ ^]^ The detailed polymerization results show a similar trend to the above‐reported for LA polymerization. It is noteworthy that **C1** catalyzes the polymerization of CL to full conversion within a few seconds, yielding PCL with a molar mass of up to 127 000 g mol^−1^. The catalysts developed by Mazzeo (**CC3**) and Lamberti (**CC4**) et al. can also catalyze lactide and caprolactone polymerization (Figure 3), although a clear difference in reaction speed between lactide and caprolactone can be observed here.^[^ ^68^, ^70^ ^]^ In contrast to **C1**, which has similar reaction times, a longer reaction time is required for caprolactone polymerization with the catalysts **CC3** and **CC4**. It should be noted that Lambert et al. polymerizes at room temperature in solution (THF) (**CC4**: 100:1:1, 20 °C, 420 min, X_NMR_ = 80%, M_n, exp_ = 10 100 g mol^−1^, *Ð* = 1.19, (Figure 3), making a direct comparison difficult. The comparison of the polymerization details of the fastest zinc triflate bisguanidine catalyst so far (**CC2**: 2000:1, 53 min, 53% conversion, *M*_n_  =  107 000 g mol^−1^, *Ð*  =  1.5, (Figure 3) shows that an enormous increase in reaction time with simultaneously significantly higher conversion is achieved with the new catalyst **C1** presented in this study (Table S8, Supporting Information, entry 1).^[^ ^55^ ^]^ A comparison with the zinc triflate hydroquinoline catalyst ([Zn(TMGhydroqu)_2_](OTf)_2_, similarly active in lactide polymerization and known from literature) shows that the catalyst is inactive in CL polymerization and illustrates the extraordinarily high activity of **C1**.^[^ ^54^ ^]^ The structural parameters of the complexes might provide a possible explanation. The bond length of the Zn─N_amine_ bond is slightly longer compared to the known complex, which could facilitate the nucleophilic attack on the CL. The biggest difference and the explanation for the high activity of **C1** can be explained by sterics. A second guanidine unit has a significantly higher steric hindrance compared to the NMe_2_ group of **C1**, which ensures better accessibility for the monomers. Compared to the hydroqu‐unit of catalyst [Zn(TMGhydroqu)_2_](OTf)_2_, **C1** has been designed with a higher flexibility of the ligand backbone. The structural parameters of **C1** are very comparable to the well‐known zinc triflate bisguanidine complex **CC2**, which differs mainly in the tetrahedral distortion. This complex also catalyzes the CL polymerization (0.0487 L mol^−1^ s^−1^), slower, suggesting that a slightly shorter bond length with an accompanying smaller angle has a negative influence on the accessibility of the monomer and thus also an inhibition of the activity. The literature comparison with the selected catalysts **CC1** – **CC4** (Figure 3) demonstrates that the catalyst **C1** presented here has an activity of the same order of magnitude. The catalytic activity in CL polymerization is particularly noteworthy.

To further elucidate the origin of the higher activity of **C1** compared to the structurally similar catalyst **CC2**, density functional theory (DFT) studies were carried out. Therein, the cationic structures of the respective catalysts as well as the first zinc‐coordinated lactone intermediate (for computational details, see Supporting Information) were investigated. Analyses of electronic parameters for the cationic species **C1** and **CC2** through Hirshfeld Population Analysis (HPA)^[^ ^71^, ^72^, ^73^, ^74^, ^75^, ^76^ ^]^ showed a larger partial charge on the zinc for **C1** (+0.355 e^−^) than for **CC2** (+0.346 e^−^) (Table S12, Supporting Information). This indicates a higher Lewis acidity of **C1**. Consequently, this correlates with the higher activity. When a lactide molecule approaches the catalyst, the difference in the partial charges between **C1** and **CC2** becomes negligible. However, steric parameters obtained with DBSTEP^[^ ^77^ ^]^ show less coordinative hindrance of the lactide for **C1** compared to **CC2**. This is visible in the Buried Volume on the carbonyl oxygen of the lactide (57.2% for **C1** and 62.8% for **CC2**). Therein, steric influences are crucial to describe the higher activity of **C1**, as the coordination is facilitated by the larger empty space at the active site (Figure S23, Supporting Information). For CL, the Hirshfeld partial charge is comparably higher for **C1** (+0.401 e^−^) to **CC2** (+0.358 e^−^), corresponding to a larger Lewis acidity and activity. This is further emphasized by the steric parameters, as the Buried Volume for **C1** is smaller (55.6%) compared to **CC2** (61.7%). Thus, the zinc center of **CC2** is more shielded, giving limited access for coordination of the CL molecule due to the bulky guanidine. This is substituted by a sterically less demanding NMe_2_ group in **C1**. Hence, the extraordinary activity of **C1** toward CL is a result of its relatively low steric hindrance in combination with the high electrophilicity of the zinc center in the presence of CL.

To further investigate whether the polymerization follows the coordination‐insertion mechanism or a cationic mechanism, the atomic Hirshfeld partial charges of the zinc‐coordinated lactide intermediate of **C1** are compared to the partial charges of neutral complex **C2** and a fictitious system that comprises only a zinc(II) cation and the lactide in its coordination sphere (Table S13, Supporting Information).^[^ ^78^, ^79^ ^]^ Herein, the former represents a catalyst that is most likely following the coordination‐insertion mechanism, whereas the latter (Zn‐LA) is a model for the coordination of a cationic mechanism. The similarity in Hirshfeld partial charges on both the lactide oxygen atom (−0.233 e^−^ for **C1**, −0.253 e^−^ for **C2**) and the attacking guanidine nitrogen donor (−0.211 e^−^ for **C1**, −0.198 e^−^ for **C2**) between the intermediates of system **C1** and the neutral system **C2** suggests a mechanistic analogy. In contrast, the cationic pathway would involve a substantial negative charge build‐up on the oxygen atom of lactide (−0.282 e^−^), which is not observed. This supports the polymerization via the coordination‐insertion mechanism using **C1** as a catalyst. In addition, matrix‐assisted laser desorption ionization time‐of‐flight (MALDI‐ToF) MS end group analyses were also performed. Short‐chain polymers with an excess of co‐initiator were produced and analyzed. The results are shown in the supporting information.

### 2.2 (Bio)Polyester Degradation Studies

For a circular plastics economy, it is very important to consider the EoL scenario of a polymer material. Therefore, in addition to the polymerization of bio‐based monomers, the activity in recycling is also important. A multifunctional catalyst for both, the synthesis and chemical recycling of (bio)polyesters is therefore highly desirable. Hence, **C1** was examined in more detail in the chemical recycling of polyesters. The alcoholysis of PLA to lactic acid esters (lactates) is an open‐loop recycling process from which new platform chemicals, green solvents or starting materials for recovering the monomer are generated. The reaction can proceed via two pathways: In solution, the irreversible formation of oligomers usually takes place, which further degrade reversible to the corresponding alkyl lactates. The random scission of the polymer chain follows a pseudo‐first‐order mechanism. Another possible degradation pathway is scission at the chain end of the polymer with direct formation of the alkyl lactate. This mechanism is more likely to occur if no additional solvent is used.^[^ ^49^ ^]^ The course of the reaction was monitored via ^1^H NMR spectroscopy of the methine group signals (Figure S18, Supporting Information), according to literature.^[^ ^66^ ^]^ By integrating the resonances in the ^1^H NMR spectrum, the conversion (*X*) of the internal methine groups of PLA, the selectivity (*S*) of the alkyl lactate, and the yield (*Y*) of the product can be calculated.

**Figure 5:**
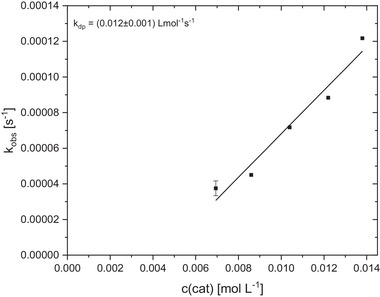
Plot of *k*_obs_ against the catalyst concentration for the depolymerization of PLA (250 mg) at 60 °C in THF using **C1** at catalyst loadings between 1.0 mol% and 2.0 mol% (regarding the polymer ester bond) and a fixed MeOH loading of 7 eq. The methanolysis straight line is not a straight line of origin due to a small extent of catalyst deactivation.

For an exact comparison of the catalyst activity, kinetic studies of the PLA methanolysis were performed at different catalyst loadings. As described for the ROP, the rate constant of depolymerization *k*_dp_ can be determined by plotting *k*_obs_ (reaction conditions: 250 mg PLA (*M*_n_  =  45 000 g mol^−1^, film produced by bio‐mi Croatia (Ltd.)), 1 mL MeOH, catalyst loading of 1 mol% to 2 mol% (based on the polymer ester bond),4 mL THF, 60 °C) against the catalyst concentration and allows a concentration‐independent comparison with catalyst systems known from literature. From this, a *k*_dp_ value of 0.012 L mol^−1^ s^−1^ is determined for **C1** (**Figure** **5**). Compared to the fastest zinc triflate bisguanidine catalyst so far (*k*_dp_  =  0.188 L mol^−1^ s^−1^), **C1** is about one order of magnitude slower.^[^ ^57^ ^]^ This result is contrary to expectations, since previously effective polymerization catalysts are also highly active in recycling.^[^ ^60^ ^]^ Table S10 (Supporting Information, entries 1–5) lists the detailed degradation results, which illustrate that no full conversion is achieved with a catalyst loading of 1 mol%, even after 48 h. A twofold increase in the catalyst concentration results in almost complete conversion after 6 h. Compared to the industrially used polymerization catalyst Sn(Oct)_2_ (*k_o_*_bs_  =  6.30 × 10^−5^ s^−1^), **C1** with 3.83 × 10^−5^ s^−1^ is in the same order of magnitude.^[^ ^80^ ^]^ Ethanolysis under the same reaction conditions also shows a PLA conversion of 73% after 24 h with an ethyl lactate yield of 39% (Table S11, Supporting Information, entry 7). The side products are the α‐ and Ω‐oligomers, forming due to the reaction mechanism by random chain scission (Figure S18, Supporting Information).

In the next step methanolysis without solvent at 150 °C was tested. The adjustment of the reaction conditions (150 °C, solvent‐free, 250 mg PLA, 1 mol% **C1** (based on the polymer ester bond), 7.1 eq alcohol, 260 rpm) shows an enormous increase in activity of the catalyst and within 15 min PLA is completely converted with a product yield of 94% (Table S11, Supporting Information, entry 6). The control experiment without a catalyst (150 °C, 7.1 eq MeOH, 260 rpm) yielded only a minimal conversion of 8% after 24 h, with a selectivity regarding MeLa formation of 7% and a MeLa yield of 1%. Thus, **C1** is highly active in the solvent‐free depolymerization of PLA to the alkyl lactates (methyl and ethyl lactate). Based on the obtained results, the primary alcohols *n*‐butanol (*n*‐BuOH), *iso*‐butanol (*i*‐BuOH), and benzyl alcohol (BnOH) with different steric demands were also investigated in the alcoholysis of PLA (Table S11, Supporting Information, entries 9–12), resulting in a wide range of value‐added products. With the alcohols *n*‐BuOH and *i*‐BuOH, full conversion and yield could be achieved after 30 min. The sterically more demanding alcohol BnOH reached a conversion and yield of 72% at the same time. It should be noted that the reaction does not proceed via oligomer formation; instead the degradation of PLA takes place directly via the chain end of the polymer. This is a well‐known result from the literature and has already been demonstrated with catalyst systems from our working group.^[^ ^57^, ^67^, ^80^ ^]^ Comparison with catalysts known from literature for PLA alcoholysis is difficult, as no standardized method exists for testing the activity of new catalysts. As a consequence, reaction conditions such as alcohol loading or temperature vary significantly, allowing only an approximate classification. In 2025, Jones and coworkers tested a series of zinc {ONO} complexes in the methanolysis of PLA with a catalyst loading of 8 wt.% (0.5–1.5 mol%) at 80 °C in THF for 8 h and achieved PLA conversions of 70–100%.^[^ ^81^ ^]^ In addition to open‐loop recycling, closed‐loop recycling is also of great importance, as the monomer can be recovered directly and reused in the polymerization process. Recycling catalyzed with **C1** shows a conversion of PLA to LA of 79% after 24 h (250 mg PLA, 11.7 wt.% C1, 150 °C, 24 h, vacuum, Figure S21, Supporting Information). Using a sublimation equipment, an isolated yield of 50% can be determined (Figure S22, Supporting Information). The isolated lactide has an optical rotation of ${\left[\alpha \right]}_{D}^{\mathrm{rt}}$ = −240 (c = 1 in toluene), indicating a low degree of epimerization (9% *meso*‐lactide, recryst. l‐LA. ${\left[\alpha \right]}_{D}^{\mathrm{rt}}$ = −284 (c = 1 in toluene, this value serves as a comparison. PLA was commercially purchased, with a 96% purity of l‐lactide.).

However, our plastic consumer goods often consist of more than just a single type of polymer. For specific applications, different polymers are combined with each other and copolymers or polymer blends are produced, which are often further enhanced by additives, making recycling difficult.^[^ ^82^ ^]^ In traditional mechanical recycling, this is similar to downcycling, as the quality of the material obtained is limited.^[^ ^83^ ^]^ Chemical recycling provides a solution, as this recycling method enables the selective degradation of one type of polymer, and the end products are more versatile.^[^ ^84^ ^]^ Due to improper separation processes in plastic waste management, PLA is one of the most frequently detected plastics in recycled PET.^[^ ^85^, ^86^ ^]^ In addition, PCL is often used for PLA blends to design the material properties for special applications.^[^ ^87^, ^88^ ^]^ Therefore, **C1** was tested in the depolymerization of PCL and PET. We observe that the catalyst is also highly active in the methanolysis and ethanolysis of PCL, reaching a yield of 60% methyl 6‐hydroxyhexanoate (MeHex) or 80% ethyl 6‐hydroxyhexanoate (EtHex) after 60 min at 150 °C (Table S11, Supporting Information, entries 13–14). Due to the high activity of **C1**, the degradation of a PLA/PCL blend was tested in the next step. For this purpose, 11.7 wt% catalyst was used, and the solvent‐free conditions were chosen (Table S11, Supporting Information, entries 15–16). First, the selective degradation of PLA to the corresponding alkyl lactate (100% conversion and yield of MeLa and EtLa) takes place, and a low degradation of PCL (conversion and yield of 18% MeHex and 14% EtHex) can be monitored after 60 min. The most likely cause of the observed difference in reactivity compared with pure PLA and PCL is the polymer structure of the blend component, which has already been reported in the literature.^[^ ^67^ ^]^ The PET degradation was finally tested via methanolysis at 150 °C and glycolysis at 180 °C with **C1** (2.5 mol%), based on the synthesis procedure described in the literature by Jones et al. After 3 h and 4 h, respectively, colorless crystals were isolated, and a yield of 55% dimethyl terephthalate (DMT) and 76% bis(hydroxyethyl) terephthalate (BHET) was obtained. These products can be used as monomers for the polymerization of PET, which corresponds to closed‐loop recycling. The comparison with the zinc guanidine catalysts known from literature shows that the catalyst **C1** presented here is highly active in the recycling of PET and achieves higher yields of the monomers in a shorter reaction time.^[^ ^57^, ^80^ ^]^

**Figure 6:**
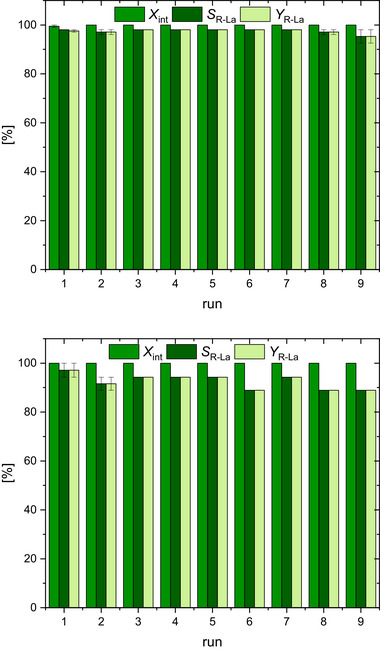
PLA conversion (*X*_int_), selectivity toward alkyl lactates (*R*‐La) formation (*S_R_*_‐La_), and *R*‐La yield (*Y_R_*_‐La_) for the solvent‐free PLA alcoholysis with 1 mol% **C1** (regarding the polymer ester bonds) with fixed MeOH (above) and EtOH (bottom) loading of 7 eq at 150 °C for 1 h reaction time with subsequent in situ catalyst recycling. The experiments were performed in duplicate. If there is no error bar, there is no error deviation within the results.

Another important aspect in industrial processes is the recyclability of the catalyst. This has already been described in detail in the literature, e.g., by Palkovits and coworkers.^[^ ^89^ ^]^ **Figure** **6** shows the in situ catalyst recycling for the solvent‐free methanolysis (top) and ethanolysis (bottom) processes at 150 °C after 1 h reaction time, respectively. The first runs show 100% PLA conversion with almost 100% yield (methyl lactate and ethyl lactate, respectively). After each run, the product and remaining alcohol were removed under reduced pressure. Subsequently, PLA (250 mg) and the corresponding alcohol (7.1 eq) were again added to the catalyst residue. A total of nine cycles was carried out, and no significant loss of activity was noted. After the sixth run, only a small drop of ≈10% can be detected in the ethyl lactate yield. Catalyst recycling in methanolysis was found to be almost at a constant level of high performance. Compared to zinc catalysts known from literature, **C1** is extremely active in catalyst recycling and shows for the first time, the highest recyclability with 9 runs without loss of activity. Other zinc guanidine catalysts from our working group show a significant loss of activity after the 4th cycle.^[^ ^57^, ^80^ ^]^ Thus, it could be demonstrated that **C1** is not only highly active in the chemical recycling of polyesters but retains its activity even when applied under these harsh reaction conditions.

## 3 Conclusion

In conclusion, we were able to present and fully characterize two new zinc hybrid guanidine ligands and six new zinc hybrid guanidine complexes. **C1** was identified to be particularly active in LA and CL polymerization under industrially relevant bulk conditions, outperforming by far the activity of the industrially used toxic Sn(Oct)_2_ catalyst. For the first time, propagation rate constants (*k*_p_) of the same order of magnitude were obtained for LA and CL ROP, underlining the exceptionally high activity of this catalyst. The resulting colorless polymers PLA (118 000 g mol^−1^) and PCL (127 000 g mol^−1^) were synthesized with high molar masses. Furthermore, the solvent polymerization of LA was also successful and led to enhanced molar mass control.

**C1** was also tested in the chemical recycling of polyesters, whereby particularly high activities were achieved under solvent‐free conditions at 150 °C. PLA was recycled with different sterically demanding alcohols to the corresponding alkyl lactates, which can be used as platform chemicals, green solvents or as starting materials for monomer synthesis. After reaction times of 15 to 75 min, conversions of 88 to 100% were achieved. Outstanding activity was shown with **C1** in catalyst recycling, whereby 9 recycling runs were carried out without any significant loss of activity. In addition, the degradation of PCL and PET, as well as the selective PLA recycling from a PLA‐PCL‐blend, were also successful, which underlines the versatility of the catalyst presented in both open‐ and closed‐loop recycling.

Therefore, this study paves the way to a non‐toxic catalyst alternative with a holistic view of our limited resources. A catalyst as an all‐round talent catalyzing both biopolyester polymerization and depolymerization, and thus getting closer to a circular plastics economy.^[^ ^90^, ^91^, ^92^, ^93^, ^94^, ^95^, ^96^, ^97^, ^98^, ^99^, ^100^, ^101^, ^102^, ^103^, ^104^, ^105^, ^106^, ^107^, ^108^, ^109^, ^110^, ^111^, ^112^, ^113^, ^114^, ^115^, ^116^, ^117^, ^118^, ^119^, ^120^, ^121^, ^122^, ^123^, ^124^, ^125^, ^126^, ^127^ ^]^

## Conflict of Interest

The authors declare no conflict of interest.

## Supporting information



Supporting Information

## Data Availability

The data that support the findings of this study are openly available in RADAR4Chem at https://radar4chem.radar‐service.eu/radar/en/dataset/kr7x78jn6hcbu6w8.
