# Supplementary material for: All‐Round Talent: Unique Zinc Guanidine Catalyst Performs Efficiently in Synthesis and Chemical Recycling of (Bio)Polyesters
Source: Adv Sci (Weinh). 2025 Oct 24;13(8):e11260. doi: 10.1002/advs.202511260 (PMC12884724; doi:10.1002/advs.202511260)
Supplement: Supplementary file 1 — Supporting Information [file ADVS-13-e11260-s001.docx]

Supporting Information

**All-round Talent: Unique Zinc Guanidine Catalyst Performs Efficiently in Synthesis and Chemical Recycling of (Bio)Polyesters**

*Tabea Becker, Koki Takeuchi, Carolin Süßmuth, Frederico Boekhoff, Martin A. Schäfer, Dr. Shunsuke Kato, Dr. Alexander Hoffmann, Prof. Dr. Takashi Hayashi, Prof. Dr. Sonja Herres-Pawlis*

**Supporting Information**

Table of Content

[1. General Information and Methods 1](#_Toc208583736)

[1.1 NMR spectroscopy 1](#_Toc208583737)

[1.2 Mass spectrometry 1](#_Toc208583738)

[1.3 Infrared spectroscopy 1](#_Toc208583739)

[1.4 Elemental analysis 1](#_Toc208583740)

[1.5 X-ray diffraction analysis 2](#_Toc208583741)

[1.6 Thermogravimetric analysis 2](#_Toc208583742)

[1.7 Size exclusion chromatography 2](#_Toc208583743)

[1.8 *in situ* Raman spectroscopy 2](#_Toc208583744)

[1.9 Differential Scanning Calorimetry (DSC) 2](#_Toc208583745)

[1.10 Matrix Assisted Laser Desorption Ionization —Time of Flight Mass Spectrometry (MALDI-ToF-MS) 3](#_Toc208583746)

[1.11 Polarimetry 3](#_Toc208583747)

[1.12 Computational Details 3](#_Toc208583748)

[2. Ligand synthesis and characterization 3](#_Toc208583749)

[2.1 (R,R)TMGNMe_2_(1,2)ch (L1) 4](#_Toc208583750)

[2.2 (R,R)DMEGNMe_2_(1,2)ch (L2) 4](#_Toc208583751)

[3. Complex synthesis and characterization 5](#_Toc208583752)

[3.1 [Zn{(*R*,*R*)TMGNMe_2_(1,2)ch}_2_](OTf)_2_ (C1) 5](#_Toc208583753)

[3.2 [ZnCl_2_(*R,R*)TMGNMe_2_(1,2)ch] (C2) 5](#_Toc208583754)

[3.3 [ZnBr_2_(*R,R*)TMGNMe_2_(1,2)ch] (C3) 6](#_Toc208583755)

[3.4 [Zn{(*R,R*)DMEGNMe_2_(1,2)ch}_2_](OTf)_2_ (C4) 6](#_Toc208583756)

[3.5 [ZnCl_2_(*R,R*)DMEGNMe_2_(1,2)ch] (C5) 7](#_Toc208583757)

[3.6 [ZnBr_2_(*R,R*)DMEGNMe_2_(1,2)ch] (C6) 8](#_Toc208583758)

[4. Crystallographic Data 9](#_Toc208583759)

[5. Polymerization Details 12](#_Toc208583760)

[5.1 l-Lactide polymerization in bulk 16](#_Toc208583761)

[5.2 l-Lactide polymerization in solution 18](#_Toc208583762)

[5.3 ε-Caprolactone polymerization 19](#_Toc208583763)

[6. Chemical recycling 21](#_Toc208583764)

[7. Computational results 26](#_Toc208583765)

[References 27](#_Toc208583766)

# 1. General Information and Methods

All experiments were performed under nitrogen atmosphere (99.999%), which was dried with P_4_O_10_ granulate, using standard Schlenk techniques, unless otherwise stated. The glassware was heated under vacuum (3 × 15 min) or placed in a drying oven at 150 °C overnight and cooled under a nitrogen atmosphere. The solvents used were purchased in technical purity, dried according to standard procedures, and kept under nitrogen before use.^[1]^ THF_abs_ was used for catalyst synthesis and depolymerizations. Zn(OTf)_2_ was recrystallized in THF_abs_. The *N*,*N,N*`*,N*`-Tetramethylchloroformamidinium chloride (TMG Vilsmeier salt) *N*,*N`-*Dimethylethylenechloroformamidinium chloride (DMEG Vilsmeier salt) was provided by Prof. Dr. Sonja Herres-Pawlis and Dr. Alexander Hoffmann, synthesized after known literature procedures and stored under nitrogen atmosphere.^[2,3]^ l-Lactide PURALACT®L polymer grade (water < 0.01%, free acid = 0.54 meq kg^−1^) was donated by Total Corbion PLA, recrystallized in toluene_abs_ and stored under nitrogen. ε-Caprolactone was dried over CaH_2_ for 24 h at room temperature, distilled under reduced pressure and stored under nitrogen. The polymer and polymer blend films for degradation studies were donated by bio-mi Ltd. (Croatia). All chemicals were purchased from abcr GmbH, Acros Organics, Carl Roth GmbH & Co. KG, Grüssing GmbH, Sigma Aldrich, TCI Deutschland GmbH, TH. Geyer GmbH & Co. KG, VWR International and were used without further purifications, unless otherwise stated.

## 1.1 NMR spectroscopy

NMR spectra were recorded on a Bruker Avance III HD 400 or a Bruker Avance II 400 nuclear resonance spectrometer at 25 °C. Resonances were referenced to the residual solvent resonance, relative to TMS (^1^H-NMR: CDCl_3_: *δ* = 7.26 ppm, DMSO-*d6*: *δ* = 2.50 ppm, DCM-*d2*: *δ* = 5.32 ppm; ^13^C-NMR: CDCl_3_: *δ* = 77.16 ppm, DMSO-*d6*: *δ* = 39.52 ppm, DCM-*d2*: *δ* = 53.84 ppm). Data in the ^1^H and ^13^C NMR spectra are stated as follows: chemical shift (δ in ppm) (multiplicity, coupling constant (J in Hz), integration). To describe the multiplicity of the signals the following abbreviations are used: s = singlet, d = doublet, t = triplet, q = quartet, m = multiplet or combinations thereof. For a more detailed analysis of the resonances various 2D NMR experiments (COSY, HSQC, HMBC) were conducted. Hydrogen and carbon atoms of all molecules are consecutively numbered for a clear and simple classification. For the Bruker Avance III HD 400 the software Topspin (Version 3.5 pl 7) from Bruker and for the Bruker Avance II 400 the software TopSpin (Version 2.1) from Bruker were used for data acquisition. For visualization and examination of the NMR spectra the software MestReNova (Version 14.2.3-29241) from Mestrelab Research was used.

## 1.2 Mass spectrometry

Electron spray ionization (ESI) high-resolution mass spectra and the APCI mass spectra were performed on an UHR-TOF Bruker Daltonik maXis II, an ESI-quadrupole time-of-flight (qToF) mass spectrometer capable of a resolution of at least 80.000 FWHM, which was coupled to a Bruker Daltonik ESI ion source. Detection was either in positive or in the negative ion mode. The mass spectrometer was calibrated subsequently to every experiment via direct infusion of a L proline sodium salt solution, which provided a m/z range of singly charged peaks up to 3000 Da in both ion modes. Or with a ThermoFisher Scientific LTQ Orbitrap XL. The source voltage was 4.49 kV, the capillary temperature amounted to 299.54 °C. The tube lens voltage lay between 110 and 130 V.

## 1.3 Infrared spectroscopy

FT-IR spectra were recorded with a Shimadzu IRTracer 100 using a CsI beam splitter in combination with an ATR unit (Quest model from Specac utilizing a robust monolithic crystalline diamond) in a 2 cm^-1^ resolution. For data acquisition, the software LabSolution IR (Version 2.15) from Shimadzu was used. The obtained IR data are stated as reciprocal wave numbers of the absorption (cm^-1^). The vibration bands of the spectra are characterized based on the Würzburger Model as follows: vw = very weak, w = weak, m = medium, s = strong and vs = very strong.

## 1.4 Elemental analysis

Elemental analysis was conducted with an elementar vario EL or an elementar vario EL cube.

## 1.5 X-ray diffraction analysis

The single crystal diffraction data for **C1**–**C6** were collected with a four-circle goniometer Stoe Stadivari with Dectris Pilatus3 R 200 K hybrid pixel detector using Geni 3D high flux Mo-Ka radiation (λ = 0.71073 Å: **C1**, **C4** and **C5**) or Cu-Ka radiation (λ = 1.54186 Å: **C2**, **C3** and **C6**) at 100 K. Temperature control was achieved with an Oxford Cryostream 800. Crystals were mounted with grease on glass fibers. Data were collected with X-Area Pilatus and integrated with X-Area Integrate and X-Area Recipe.^[4–6]^ The absorption correction was performed by Gaussian integration with Stoe X-Red32, afterwards scaling of reflections with X-Area LANA.^[7]^ The single crystal diffraction data for **C1**–**C6** are presented in Tables S1–S3. The structures were solved by direct and conventional Fourier methods and all non-hydrogen atoms were refined anisotropically with full-matrix least-squares based on F^2^ (XPREP, SHELXS-97 or SHELXT and ShelXle).^[8–11]^ Hydrogen atoms were derived from difference Fourier maps and placed at idealised positions, riding on their parent C atoms, with isotropic displacement parameters Uiso(H) = 1.2 Ueq(C) and 1.5 Ueq(C methyl). All methyl groups were allowed to rotate^[8]^ but not to tip.

**C4** is a twin and the data set was treated with the TwinRotMat routine as implemented in Platon (twofold rotation axis along [ 1 0 2] and twin law -1 0 0 0 -1 0 1 0 1 with BASF= 0.25383).^[12,13]^

Full crystallographic data have been deposited with the Cambridge Crystallographic Data Centre as supplementary no. CCDC – 2434180 for **C1**, CCDC – 2434181 for **C2**, CCDC – 2434182 for **C3**, CCDC – 2434183 for **C4** CCDC – 2434184 for **C5** and CCDC – 2434185 for **C6**. Copies of the data can be obtained free of charge on application to CCDC, 12 Union Road, Cambridge CB2 1EZ, UK (fax: (+44)1223-336-033; e-mail: deposit@ccdc.cam.ac.uk).

## 1.6 Thermogravimetric analysis

The thermogravimetric analysis (TGA) of the complexes was carried out with a Netzsch Simultaneous Thermal Analyzer (Type 449F5) in a temperature range of 25–150 °C and 25–200 °C with a heating rate of 5 °C min^-1^, holding time of 120 min and subsequent temperature increase to 1000 °C with a heating rate of 5°C min^-1^.

## 1.7 Size exclusion chromatography

The average molar masses and the mass distributions of the used PLA samples were determined by size exclusion chromatography (SEC) on a Viscotek GPCmax VE-2001 applying a flow rate of 1 mL/min at 25 °C with THF as the mobile phase. The device was equipped with a HPLC pump, two Malvern Viscotek T columns (porous styrene divinylbenzene copolymer) with maximum pore size of 500 and 5000 Å, a refractive index detector (VE-3580) and a viscometer (Viscotek 270 Dual detector). The software Omnisec – 5.12 was used for data acquisition. The results were evaluated using conventional calibration (polystyrene) with Mark-Houwink correction factor of 0.56 for PCL and 0.58 for PLA.^[14,15]^

## 1.8 *in situ* Raman spectroscopy

Raman spectra were measured under process conditions (150 °C, Ar atmosphere) using a RXN1 spectrometer of Kaiser Optical System with a 785 nm laser. The used detector is a TE-Cooled, 1024 CCD detector and the used stirrer corresponds to the model PRE1946 of Premex Reactor AG with a torsional moment of 20 Ncm. An immersion probe with a sapphire lens (d = 0.1 mm) was applied to the autoclave optimized for monitoring of biphasic reaction mixtures.^[16]^ The enclosure and heat sink of the laser were controlled with the program Invictus Laser Control v1.6.0.1. The data of the measurement were collected with i.C. Raman 4.1 and the obtained time-resolved data were processed with the PEAXACT 5.0 software. Kinetic data were obtained by integration of characteristic peaks of the Raman spectrum. For the polymerization of lactide the lactide peak (656 cm^−1^) and the polylactide peak (872 cm^−1^) were integrated. For the ε-caprolactone polymerization the characteristic monomer peaks at 733 cm^−1^ and 696 cm^−1^ were integrated.

## 1.9 Differential Scanning Calorimetry (DSC)

DSC curves of selected polymer samples were recorded on a Netzsch DSC 204 F1 Phoenix equipped with an intra-cooler. The samples were weighed into 50 µL aluminum pans and sealed with punctuated aluminum lids. For all measurements, four cycles were performed, starting at 20 °C and subsequent heating to 200 °C. After each cooling, an isotherm at 20 °C was applied for 15 min. After the first heating, an isotherm at 200 °C was applied for 3 h. A heating rate of 10 K min^–1^ and a nitrogen flow of 40 mL min^–1^ was applied. The analysis of data was performed with the software NETZSCH Proteus – Thermal Analysis.^[17]^

## **1.10 Matrix Assisted Laser Desorption Ionization** —Time of Flight Mass Spectrometry (MALDI-ToF-MS)

The end group analysis was performed by MALDI-TOF on a Bruker ultrafleXtreme equipped with a 337 nm smart beam laser in the reflective mode. THF solutions of trans-2-[3-(4-tert-butylphenyl)-2-methyl-2-propenylidene]malononitrile (DCTB) (5 μL of a 20 mg/mL solution), sodium trifluoroacetate (0.1 μL of a 10 mg/mL solution), and analyte (5 μL of a 10 mg/mL) were mixed and a droplet thereof applied on the sample target. For spectra 4000 laser shots with 24% laser power were collected. The laser repetition rate was 1000 Hz. The homopolymer analysis was performed using Polymerix software (Sierra Analytics).

## 1.11 Polarimetry

The optical rotation value was determined using a P3000 digital polarimeter from A. KRÜSS Optronic GmbH. The polarimeter is using a wavelength of 589 nm (LED with filter). For the measurements a glass cuvette of 10 cm was used.

1.12 Computational Details Based on the crystal structures of **C1** as well as **CC2**, preoptimizations were performed using the semiempirical electron structure method GFN2-xTB^[18]^ implemented in the program xtb (version 6.6.2)^[19]^. Within preoptimiza-
tions, the implicit solvation model analytical linearized Poisson-Boltzmann (ALPB)^[20]^ for THF was
used. To obtain the subsequent coordinated intermediate, docking of the respective complex and lactone (L-LA or CL) was carried out using xtbiff (version 1.1). Orbitals required for docking were acquired using the linearized molecular orbital (LMO) approach via the GFN1-xTB method.^[21]^ For comparison, the Zn-O_carbonyl,lactone_ bond length was constrained to 3.8 Å (force constant was set to 1 E_h_/Bohr^2^). Conformer sampling was carried out using CREST^[22]^ (version 2.11.3) based on the GFN-FF level of theory^[23]^. Resampling occurred through reoptimization of the conformers on the GFN2-xTB level of theory. Both computational steps were performed using the above-mentioned constraints as well as the ALPB solvation model for THF. The 20 lowest lying conformers (clustered with the CREGEN routine in CREST) were then optimized using density functional theory (DFT) using ORCA (version 6.0.0)^[24]^. Herein, the hybrid density functional TPSSh^[25]^ along with the Ahlrichs type basis set def2-TZVP^[26]^ was utilized. As empirical dispersion correction, the D4 dispersion model was used.^[27,28]^ During DFT calculations, implicit solvation was treated with the conductor-like polarizable continuum model (CPCM) for THF.^[29]^ The re-solution-of-the-identity (RI) approximation was used for Coulomb integrals and the numerical chain-of-sphere integration for the Hartree-Fock exchange integrals (COSX)^[30,31]^ was used for calculation acceleration. The applied auxiliary base was def2/J.^[32]^ Geometry optimization was carried out using either a tight or very tight convergence criterion (10^-9^ a.u.), and the above-mentioned constraints for the coordinated intermediate. After geometry optimization, frequency calculations were carried out to check for minima. For the coordinated intermediates of **CC2**, and imaginary frequency of 18.16 cm^-1^ for the LA species and -14.60 cm^-1^ for the CL species were found respectively. These correspond to rotations of methyl groups. Based on the final geometry, Hirshfeld population analysis (HPA) was performed at the DFT level of theory.^[33–38]^ Steric parameters were computed via the tool DBSTEP.^[39]^ A Radius of 4 Å was applied during the calculation. In addition, the zinc-coordinated lactide species of **C2** as well as the fictitious zinc-lactide complex were generated and optimized analogously. For the latter, a free zinc(II) ion was used instead of the crystal structure as starting structure for the docking process. Moreover, the fictitious geometry possesses two imaginary frequencies at -50.11 cm^-1^ and -12.98 cm^-1^, respectively. These are still present, even after the constrained geometry optimization was performed using extreme convergence criteria (10^-14^ a.u.). The observed low-frequency imaginary mode at –50.11 cm⁻¹ corresponds to a soft motion along the constrained Zn–O distance and does not reflect a true transition state. Similar behavior has been documented in structures with enforced geometries, where such modes are considered numerical artifacts of the constraint.^[40–42]^ Likewise, the second minor imaginary mode (–12.98 cm⁻¹) is attributed to a rocking motion (ligand tilt) of the lactide unit with respect to the Zn²⁺ coordination plane without chemical relevance.

# 2. Ligand synthesis and characterization

*Trans*-*N*1,*N*1-dimethylcyclohexane-1,2-diamine (2.845 g, 20 mmol, 1.0 eq.) and triethylamine (3.050 mL, 22 mmol, 1.1 eq.) were dissolved in acetonitrile_abs_ (50 mL) and added in a 2-neck Schlenk flask with reflux condenser and dropping funnel. A solution of TMG-Vilsmeier salt or DMEG-Vilsmeier salt (22 mmol, 1.1 eq.) in acetonitrile_abs_ (50 mL) was added dropwise with ice cooling and strong stirring. The reaction solution was refluxed for 3 h (500 rpm) using an oil bath temperature of 110 °C. After cooling to RT, an aqueous NaOH solution (22 mmol, 1.1 eq in 10 mL water) was added. The solvent and triethylamine were removed under reduced pressure. The guanidine hydrochloride was deprotonated with 50% KOH solution (aq., 25 mL) and extracted with acetonitrile (3x30 mL). The combined organic phases were purified with sodium sulfate and subsequently filtered over Celite. The solvent was removed under reduced pressure. Pentane (10 mL) was added to the product to separate insoluble salts. The product solution was filtered and the solvent was removed under reduced pressure.

## 2.1 (R,R)TMGNMe_2_(1,2)ch (L1)

Yellow-brown oil, yield 61% (2.924 g, 12.16 mmol).

^1^H NMR (400 MHz, DMSO–*d6*): δ = 3.16 (td, *J* = 9.4, 3.8 Hz, 1H, c), 2.66 (s, 6H, b), 2.54 (s, 6H, b`), 2.12 (s, 6H, g), 2.12–2.07 (m, 1H, f), 1.72–1.65 (m, 2H, d), 1.65–1.57 (m, 2H, e), 1.30–1.18 (m, 2H, e`), 1.17–1.09 (m, 2H, d`) ppm.

^13^C NMR (100 MHz, DMSO–*d6*): δ = 157.4 (a), 68.6 (f), 57.3 (c), 40.7 (g), 39.6 (b), 38.5 (b`), 34.4 (d), 25.1 (e), 24.9 (e`), 23.1 (d`) ppm.

HR-MS (ESI+, MeCN), m/z (%): calculated for [C_13_H_28_N_4_+H]^+^: 241.2387 (100); found: 241.2386 (100).

IR (ATR, ṽ) = 2924 (s, ṽ(CH_aliph_)), 2854 (m, ṽ(CH_aliph_)), 2837 (w, ṽ(CH_aliph_)), 2834 (w, ṽ(CH_aliph_)), 2815 (w, ṽ(CH_aliph_)), 2784 (w), 2779 (w), 2769 (w), 1621 (vs, ṽ(C=N_gua_)), 1493 (w), 1449 (m), 1424 (w), 1403 (vw), 1360 (s), 1296 (vw), 1271 (vw), 1235 (w), 1189 (vw), 1132 (s), 1106 (w), 1095 (w), 1057 (w), 1047 (w), 1041 (w), 1033 (w), 1004 (s), 981 (vw), 949 (vw), 922 (w), 894 (w), 872 (w), 852 (w), 829 (vw), 780 (vw), 751 (vw), 736 (vw), 599 (w), 566 (vw), 553 (w), 503 (w), 490 (vw), 463 (vw), 454 (vw), 447 (vw), 446 (vw), 439 (vw), 431 (vw), 428 (vw), 423 (vw), 421 (vw), 417 (vw), 412 (vw), 408 (vw), 405 (vw) cm^–1^.

Information on the synthesis of the compound and original analysis data files are available via Chemotion Repository: 10.14272/reaction/SA-FUHFF-UHFFFADPSC-OVLFTYYMIO-UHFFFADPSC-NUHFF-NHCNI-NUHFF-ZZZ

## 2.2 (R,R)DMEGNMe_2_(1,2)ch (L2)

Yellow-brown oil, yield 73% (3.483 g, 14.6 mmol).

^1^H NMR (400 MHz, DMSO–*d6*): δ = 3.62–3.55 (m, 1H, d), 3.17–3.09 (m, 2H, c), 3.02–2.97 (m, 2H, c`), 2.69 (s, 6H, b), 2.25 (s, 6H, h), 2.17–2.10 (m, 1H, g), 1.70–1.57 (m, 4H, e/f), 1.28–1.09 (m, 4H, e/f) ppm.

^13^C NMR (100 MHz, DMSO–*d6*): δ = 153.7 (a), 68.2 (g), 55.0 (d), 48.9 (c/c`), 41.6 (h), 36.2 (b), 34.7 (e/f), 26.9 (e/f), 24.9 (e/f), 24.1 (e/f) ppm.

HR-MS (ESI+, MeCN), m/z (%): calculated for [C_13_H_26_N_4_+H]^+^: 239.2230 (100); found: 239.2231 (100).

IR (ATR, ṽ) = 2921 (m, ṽ(CH_aliph_)), 2851 (w, ṽ(CH_aliph_)), 2826 (w), 2774 (w), 1700 (vw), 1658 (vs, ṽ(C=N_gua_)), 1477 (w), 1469 (w), 1467 (w), 1462 (w), 1457 (w), 1448 (w), 1434 (w), 1410 (w), 1375 (m), 1354 (vw), 1256 (s), 1229 (w), 1192 (w), 1158 (vw), 1150 (vw), 1140 (vw), 1116 (vw), 1094 (vw), 1058 (w), 1045 (w), 1035 (w), 1023 (m), 991 (vw), 965 (w), 953 (w), 944 (w), 918 (vw), 904 (vw), 872 (w), 851 (w), 825 (w), 777 (vw), 765 (vw), 718 (w), 644 (w), 586 (w), 572 (w), 556 (vw), 494 (vw), 489 (vw), 480 (vw), 456 (vw), 451 (vw), 421 (vw), 417 (vw), 413 (vw) cm^–1^.

Information on the synthesis of the compound and original analysis data files are available via Chemotion Repository: 10.14272/reaction/SA-FUHFF-UHFFFADPSC-XXARTQKKVB-UHFFFADPSC-NUHFF-NHCNI-NUHFF-ZZZ

# 3. Complex synthesis and characterization

Ligand (*R*,*R*)TMGNMe*_2_*(1,2)ch or (*R*,*R*)DMEGNMe*_2_*(1,2)ch (0.5 mmol, 1.0 eq. for complex **C2**, **C3**, **C5** and **C6**; 0.4 mmol, 1.0 eq. for **C1** and **C4**) was dissolved in THF (2 mL) and the zinc salt ZnCl_2_, ZnBr_2_ or Zn(OTf)_2_ (0.5 mmol, 1.0 eq. for complex **C2**, **C3**, **C5** and **C6**; 0.16 mmol, 0.4 eq. for **C1** and **C4**) was dissolved in THF (6 mL) under heating. The warm ligand solution was added to the warm zinc salt solution and was cooled to room temperature. Crystals were formed from the solution after one day, which were filtered and washed afterwards with THF and Et_2_O.

## 3.1 [Zn{(*R*,*R*)TMGNMe_2_(1,2)ch}_2_](OTf)_2_ (C1)

Colorless crystals, yield 80% (0.108 g, 0.128 mmol).

^1^H NMR (400 MHz, DCM–*d2*): δ = 3.40 (td, *J* = 10.5 Hz, 3.9 Hz 2H, c), 3.11 (s, 6H, b’’), 2.96 (c, *J* = 4.0 Hz, 12H, b), 2.90 (s, 6H, b’), 2.51 (s, 6H, g’), 2.47 (s, 6H, g), 2.27 (td, J = 11.0 Hz, 2H, f), 2.02–1.96 (m, 4H, d), 1.83–1.70 (m, 4H, e), 1.49–1.37 (m, 4H, e‘), 1.34–1.12 (m, 4H, d‘) ppm.

^13^C NMR (100 MHz, DCM–*d2*): δ = 167.8 (a), 72.5 (g), 58.0 (c/f), 45.6 (g‘),41.9 (b) 41.2 (b), 41.0 (b`‘), 40.9 (b`), 38.6 (g), 34.3 (d), 25.3 (e), 25.0 (e`), 21.0 (d`) ppm.

IR (ATR, ṽ) = 2946 (w), 2872 (vw), 1621 (vw), 1534 (s), 1482 (w), 1466 (w), 1449 (w), 1429 (w), 1410 (w), 1399 (m), 1356 (vw), 1326 (vw), 1317 (vw), 1265 (vs), 1255 (vs), 1229 (m), 1221 (s), 1159 (vs), 1156 (vs), 1109 (vw), 1098 (vw), 1083 (vw), 1063 (w), 1030 (vs), 1023 (vs), 1003 (m), 962 (w), 945 (vw), 921 (w), 896 (w), 874 (m), 854 (vw), 823 (vw), 804 (w), 775 (w), 756 (w), 743 (vw), 635 (vs), 600 (w), 593 (w), 573 (w), 536 (vw), 516 (s), 490 (vw), 484 (vw), 480 (vw), 477 (vw), 463 (vw), 441 (vw), 431 (vw), 419 (vw) cm^–1^.

HR-MS (APCI+, MeCN + MeOH), m/z (%): calculated for [C_14_H_28_N_4_O_3_F_3_S^64^Zn]^+^: 453.1125 (100), [C_13_^13^CH_28_N_4_O_3_F_3_S^64^Zn]^+^: 454.1159 (16), [C_14_H_28_N_4_O_3_F_3_^34^S^64^Zn and C_14_H_28_N_4_O_3_F_3_S^66^Zn and C_12_^13^C_2_H_28_N_4_O_3_F_3_S^64^Zn and C_14_H_28_N_4_O_2_^18^OF_3_S^64^Zn]^+^: 455.1099 (63), [C_14_H_28_N_4_O_3_F_3_S^67^Zn and C_13_^13^CH_28_N_4_O_3_F_3_S^66^Zn]^+^: 456.1114 (17), [C_14_H_28_N_4_O_3_F_3_S^68^Zn and C_14_H_28_N_4_O_3_F_3_^34^S^66^Zn and C_12_^13^CH_28_N_4_O_3_F_3_S^67^Zn]^+^: 457.1084 (41), [C_14_H_28_N_3_^15^NO_3_F_3_S^68^Zn and C_14_H_28_N_4_O_3_F_3_^34^S^67^Zn and C_14_H_28_N_4_O_3_F_3_^33^S^68^Zn and C_13_^13^CH_28_N_4_O_3_F_3_^34^S^66^Zn and C_13_^13^CH_28_N_4_O_3_F_3_S^68^Zn ^+^: 458.1110 (8), [C_14_H_28_N_4_O_3_F_3_^34^S^68^Zn and C_14_H_28_N_4_O_3_F_3_S^70^Zn and C_12_^13^C_2_H_28_N_4_O_3_F_3_S^68^Zn]^+^: 459.1077 (3); found 453.1114 (100), 454.1145 (16), 455.1087 (59), 456.1107 (17), 457.1071 (38), 458.1103 (6), 459.1065 (3).

Elemental analysis calculated [%] for C_28_H_56_F_6_N_8_O_6_S_2_Zn: C 39.83, H 6.69, N 13.27; found: C 39.09, H 6.18, N 13.02.

Information on the synthesis of the compound and original analysis data files are available via Chemotion Repository: 10.14272/reaction/SA-FUHFF-UHFFFADPSC-RWQISLWUHN-UHFFFADPSC-NUHFF-LDQSP-NUHFF-ZZZ

## 3.2 [ZnCl_2_(*R,R*)TMGNMe_2_(1,2)ch] (C2)

Colorless crystals, yield 68 % (0.129 g, 0.341 mmol).

^1^H NMR (400 MHz, CDCl_3_): δ = 3.20 (td, *J* = 10.4, 4.0 Hz, 1H, c), 3.08 (s, 3H, b), 2.82 (s, 6H, b`), 2.79 (s, 3H, b), 2.69–2.63 (m, 1H, f), 2.62 (s, 3H, g’), 2.31 (s, 3H, g), 1.97–1.84 (m, 2H, d), 1.75–1.84 (m, 2H, e), 1.30–1.21 (m, 2H, e`), 1.21–1.07 (m, 2H, d`) ppm.

^13^C NMR (100 MHz, CDCl_3_): δ = 165.9 (a), 69.1 (f), 56.9 (c), 44.7 (g‘), 41.2 (b), 40,21 (b), 39.5 (b`), 39.4 (b`), 37.8 (g), 33.2 (d), 24.9 (e/e`), 20.9 (d`) ppm.

HR-MS (ESI+, MeCN), m/z (%): calculated for [C_13_H_28_N_4_^64^Zn^35^Cl]^+^: 339.1297 (100), [C_12_^13^CH_28_N_4_^64^Zn^35^Cl]^+^: 340.1322 (14), [C_13_H_28_N_4_^66^Zn^35^Cl and [C_13_H_28_N_4_^64^Zn^37^Cl]^+^: 341.1267 (89), [C_13_H_28_N_4_^67^Zn^35^Cl and C_12_^13^CH_28_N_4_^66^Zn^35^Cl and C_12_^13^CH_28_N_4_^64^Zn^37^Cl]^+^: 342.1285 (21), [C_13_H_28_N_4_^68^Zn^35^Cl and C_13_H_28_N_4_^66^Zn^37^Cl]^+^: 343.1249 (56), [C_13_H_28_N_4_^67^Zn^37^Cl and C_12_^13^CH_28_N_4_^66^Zn^37^Cl and C_12_^13^CH_28_N_4_^68^Zn^35^Cl]^+^: 344.1266 (10), [C_13_H_28_N_4_^68^Zn^37^Cl and C_13_H_28_N_4_^70^Zn^35^Cl and C_11_^13^C_2_H_28_N_4_^68^Zn^35^Cl and C_13_H_26_N_4_^70^Zn^35^Cl]^+^: 345.1229 (13); found: 339.1282 (100), 340.1309 (15%), 341.1285 (95), 342.1272 (21%), 343.1235 (57), 344.1257 (11%) and 345.1214 (15%).

IR (ATR, ṽ) = 3005 (vw), 2995 (vw), 2970 (vw), 2940 (m), 2895 (w), 2884 (w), 2868 (w), 2862 (w), 2803 (vw), 1553 (vs), 1537 (vs), 1533 (vs), 1477 (w), 1472 (m), 1464 (w), 1452 (s), 1439 (m), 1425 (s), 1410 (m), 1396 (vs), 1381 (w), 1364 (w), 1335 (w), 1319 (w), 1256 (w), 1240 (w), 1220 (vw), 1175 (w), 1157 (s), 1142 (w), 1113 (w), 1082 (vw), 1065 (w), 1053 (vw), 1040 (w), 1028 (s), 1013 (m), 962 (w), 951 (w), 922 (m), 895 (w), 876 (s), 849 (w), 824 (vw), 802 (m), 777 (w), 743 (vw), 631 (w), 590 (m), 575 (w), 530 (w), 496 (vw), 478 (w), 444 (vw), 417 (vw) cm^–1^.

Elemental analysis calculated [%] for C_13_H_28_N_4_Cl_2_Zn: C 41.45, H 7.49, N 14.87; found: C 41.22, H 7.38, N 14.73.

Information on the synthesis of the compound and original analysis data files are available via Chemotion Repository: 10.14272/reaction/SA-FUHFF-UHFFFADPSC-MCLGULJYFK-UHFFFADPSC-NUHFF-LXMKZ-NUHFF-ZZZ

## 3.3 [ZnBr_2_(*R,R*)TMGNMe_2_(1,2)ch] (C3)

Colorless crystals, yield 66 % (0.155 g, 0.332 mmol).

^1^H NMR (400 MHz, CDCl_3_): δ = 3.21 (td, *J* = 10.3, 4.1 Hz, 1H, c), 3.11 (s, 3H, b), 2.84 (s, 6H, b`), 2.79 (s, 3H, b’’), 2.73–2.67 (m, 1H, f), 2.62 (s, 3H, g), 2.33 (s, 3H, g’), 1.97–1.82 (m, 2H, d), 1.74–1.60 (m, 2H, e), 1.33–1.25 (m, 2H, e`), 1.23–1.08 (m, 4H, d`) ppm.

^13^C NMR (100 MHz, CDCl_3_): δ = 166.0 (a), 68.9 (f), 57.1 (c), 45.2 (g), 41.9 (b), 40,3 (b), 39.5 (b‘‘), 38.4 (g‘), 33.4 (d), 24.9 (e), 24.9 (e`), 21.0 (d`) ppm.

HR-MS (ESI+, MeCN), m/z (%): calculated for [C_13_H_28_N_4_^64^Zn^79^Br]^+^: 383.0790 (64), [C_12_^13^CH_28_N_4_^64^Zn^79^Br]^+^: 384.0815 (9), [C_13_H_28_N_4_ZnBr]^+^: 385.0761 (100), [C_13_H_28_N_4_^67^Zn^79^Br and C_12_^13^CH_28_N_4_^64^Zn^81^Br and C_12_^13^CH_28_N_4_^66^Zn^79^Br]^+^: 386.0787 (19), [C_13_H_28_N_4_^68^Zn^79^Br and C_13_H_28_N_4_^66^Zn^81^Cl]^+^: 387.0741 (60), [C_13_H_28_N_4_^67^Zn^81^Br and C_12_^13^CH_28_N_4_^66^Zn^81^Br and C_12_^13^CH_28_N_4_^68^Zn^79^Br]^+^: 388.0764 (13), [C_13_H_28_N_4_^68^Zn^81^Br]^+^: 389.0729 (24); found: 383.0782 (65), 384.0808 (10%), 385.0758 (100), 386.0781 (19%), 387.0736 (59), 388.0756 (13%) and 389.0722 (25%).

IR (ATR, ṽ) = 2994 (vw), 2968 (vw), 2949 (w), 2936 (w), 2891 (w), 2886 (w), 2866 (w), 2859 (w), 2801 (vw), 1551 (vs), 1533 (vs), 1476 (m), 1470 (m), 1462 (m), 1452 (s), 1437 (m), 1425 (s), 1408 (m), 1396 (vs), 1381 (w), 1362 (m), 1333 (w), 1317 (w), 1256 (w), 1238 (w), 1173 (w), 1157 (s), 1138 (w), 1111 (w), 1086 (vw), 1065 (w), 1051 (w), 1038 (w), 1026 (s), 1011 (m), 961 (w), 949 (w), 920 (m), 895 (w), 874 (s), 847 (w), 822 (vw), 802 (m), 775 (w), 743 (vw), 631 (w), 588 (m), 575 (w), 528 (w), 492 (vw), 476 (w), 442 (vw), 415 (vw), 411 (vw) cm^–1^.

Elemental analysis calculated [%] for C_13_H_28_N_4_Br_2_Zn: C 33.54, H 6.06, N 12.03; found: C 33.56, H 6,06, N 12,00.

Information on the synthesis of the compound and original analysis data files are available via Chemotion Repository: 10.14272/reaction/SA-FUHFF-UHFFFADPSC-JUEVFIVBBA-UHFFFADPSC-NUHFF-LXMKZ-NUHFF-ZZ

## 3.4 [Zn{(*R,R*)DMEGNMe_2_(1,2)ch}_2_](OTf)_2_ (C4)

 Colorless crystals, yield 88% (0.118 g, 0.140 mmol).

^1^H NMR (400 MHz, DCM-*d2*): δ = 3.80–3.72 (m, 4H, c), 3.67 (td, 2H, *J* = 10.6 Hz, 3.7 Hz, d), 3.52–3.43 (m, 2H, c`), 3.29–3.20 (m, 2H, c`), 3.03 (s, 6H, b), 2.91 (s, 6H, b`), 2.51 (s, 6H, h), 2.36 (s, 6H, h`), 2.33 (td, 2H, J = 10.8 Hz, 2.7 Hz, g), 2.12–2.08 (m, 2H, e), 1.96–1.86 (m, 4H, e´), 1.75–1.68 (m, 2H, f), 1.38–1.20 (m, 6H, f/f`), 1.02-0.92 (m, 2H, e‘) ppm.

^13^C NMR (100 MHz, DCM-*d2*): δ = 167.5 (a), 72.1 (g), 56.8 (d), 51.1 (c),48.8 (c`), 45.7 (h), 38.4 (h`), 37.4 (b), 37.3 (b`), 33.4 (e), 25.1 (f), 24.4 (f`), 20.9 (e`) ppm.

HR-MS (APCI+, MeOH), m/z (%): calculated for [C_14_H_26_N_4_O_3_F_3_S^64^Zn]^+^: 451.0969 (100), [C_13_^13^CH_26_N_4_O_3_F_3_S^64^Zn]^+^: 452.1002 (15), [C_14_H_26_N_4_O_3_F_3_^34^S^64^Zn and C_14_H_26_N_4_O_3_F_3_S^66^Zn and C_12_^13^C_2_H_26_N_4_O_3_F_3_S^64^Zn and C_14_H_26_N_4_O_2_^18^OF_3_S^64^Zn]^+^: 453.0943 (63), [C_14_H_26_N_4_O_3_F_3_S^67^Zn and C_13_^13^CH_26_N_4_O_3_F_3_S^66^Zn]^+^: 454.0962 (17), [C_14_H_26_N_4_O_3_F_3_S^68^Zn and C_14_H_26_N_4_O_3_F_3_^34^S^66^Zn and C_12_^13^CH_26_N_4_O_3_F_3_S^67^Zn]^+^: 455.0924 (42), [C_14_H_26_N_3_^15^NO_3_F_3_S^68^Zn and C_14_H_26_N_4_O_3_F_3_^34^S^67^Zn and C_14_H_26_N_4_O_3_F_3_^33^S^68^Zn and C_13_^13^CH_26_N_4_O_3_F_3_^34^S^66^Zn and C_13_^13^CH_26_N_4_O_3_F_3_S^68^Zn ^+^: 456.0948 (8), [C_14_H_26_N_4_O_3_F_3_^34^S^68^Zn and C_14_H_26_N_4_O_3_F_3_S^70^Zn and C_12_^13^C_2_H_26_N_4_O_3_F_3_S^68^Zn]^+^: 457.0920 (3); found 451.0953 (100), 452.0978 (17), 453.0930 (58), 454.0946 (19), 455.0921 (38), 456.0958 (7), 457.1055 (9).

IR (ATR, ṽ) = 2945 (w), 2862 (vw), 1566 (s), 1524 (w), 1493 (w), 1470 (w), 1443 (vw), 1423 (w), 1408 (w), 1385 (w), 1298 (w), 1256 (vs), 1221 (s), 1194 (vw), 1175 (m), 1153 (vs), 1111 (vw), 1082 (vw), 1040 (w), 1024 (vs), 1007 (m), 961 (w), 939 (w), 908 (vw), 876 (w), 854 (vw), 827 (w), 810 (w), 781 (w), 756 (w), 739 (w), 663 (vw), 635 (vs), 596 (m), 573 (w), 538 (w), 517 (m), 488 (w), 478 (vw), 443 (vw) cm^–1^.

Elemental analysis calculated [%] for C_28_H_52_F_6_N_8_O_6_S_2_Zn: C 40.02, H 6.24, N 13.34; found: C 39.80, H 6.10, N 13.32.

Information on the synthesis of the compound and original analysis data files are available via Chemotion Repository: 10.14272/reaction/SA-FUHFF-UHFFFADPSC-ONONMGHJAR-UHFFFADPSC-NUHFF-LDQSP-NUHFF-ZZZ

## 3.5 [ZnCl_2_(*R,R*)DMEGNMe_2_(1,2)ch] (C5)

Colorless crystals, yield 75 % (0.142 g, 0.377 mmol).

^1^H NMR (400 MHz, CDCl_3_): δ = 3.68–3.65 (m, 2H, c), 3.56 (td, *J* = 10.2, 3.8 Hz, 1H, d), 3.30–3.26 (m, 2H, c`), 3.08 (s, 3H, b), 2.96 (s, 3H, b`), 2.73–2.67 (m, 1H, g), 2.63 (s, 3H, h), 2.34 (s, 3H, h`), 2.11–2.05 (m, 1H, e), 1.98–1.92 (m, 1H, e), 1.89–1.84 (m, 1H, f), 1.75–1.70 (m, 1H, f), 1.34–1.18 (m, 2H, f`), 1.17–1.07 (m, 2H, e`) ppm.

^13^C NMR (100 MHz, CDCl_3_): δ = 164.3 (a), 68.8 (g), 56.1 (d), 50,9 (c), 48.4 (c`), 44.5 (h), 37,5 (h`), 37.2 (b), 36.0 (b`), 32.5 (e), 24.9 (f), 24.6 (f), 21.1 (e) ppm.

HR-MS (ESI+, MeCN), m/z (%): calculated for [C_13_H_26_N_4_^64^Zn^35^Cl]^+^: 337.1136 (100), [C_12_^13^CH_26_N_4_^64^Zn^35^Cl]^+^: 338.1171 (14), [C_13_H_26_N_4_^66^Zn^35^Cl and [C_13_H_26_N_4_^64^Zn^37^Cl]^+^: 339.1109 (89), [C_13_H_26_N_4_^67^Zn^35^Cl and C_12_^13^CH_26_N_4_^66^Zn^35^Cl and C_12_^13^CH_26_N_4_^64^Zn^37^Cl]^+^: 340.1124 (21), [C_13_H_26_N_4_^68^Zn^35^Cl and C_13_H_26_N_4_^66^Zn^37^Cl]^+^: 341.1094 (56), [C_13_H_26_N_4_^67^Zn^37^Cl and C_12_^13^CH_26_N_4_^66^Zn^37^Cl and C_12_^13^CH_26_N_4_^68^Zn^35^Cl]^+^: 342.1108 (10), [C_13_H_26_N_4_^68^Zn^37^Cl and C_13_H_26_N_4_^70^Zn^35^Cl and C_11_^13^C_2_H_26_N_4_^68^Zn^35^Cl and C_13_H_26_N_4_^70^Zn^35^Cl]^+^: 343.1065 (13); found: 337.1124 (100), 338.1152 (13%), 339.1095 (90), 340.1118 (22%), 341.1080 (52), 342.1104 (9%) and 343.1060 (13%).

IR (ATR, ṽ) = 3009 (vw), 2994 (vw), 2970 (w), 2951 (w), 2941 (w), 2920 (w), 2891 (w), 2868 (w), 2851 (vw), 2806 (vw), 1584 (vs), 1504 (m), 1485 (w), 1472 (w), 1458 (m), 1423 (s), 1406 (m), 1383 (w), 1364 (w), 1335 (w), 1315 (w), 1283 (m), 1258 (w), 1250 (w), 1244 (w), 1196 (vw), 1182 (w), 1175 (w), 1136 (vw), 1113 (vw), 1088 (w), 1065 (vw), 1042 (w), 1032 (m), 1020 (m), 995 (vw), 978 (vw), 959 (m), 943 (w), 907 (w), 878 (m), 849 (w), 829 (w), 802 (w), 781 (w), 737 (w), 662 (w), 604 (w), 586 (s), 532 (w), 490 (w), 476 (w), 442 (w) cm^–1^.

Elemental analysis calculated [%] for C_13_H_26_N_4_Cl_2_Zn: C 41.68, H 7.00, N 14.95; found: C 41.68, H 6.51, N 14.94.

Information on the synthesis of the compound and original analysis data files are available via Chemotion Repository: 10.14272/reaction/SA-FUHFF-UHFFFADPSC-GBZPYVWFOZ-UHFFFADPSC-NUHFF-LXMKZ-NUHFF-ZZZ

## 3.6 [ZnBr_2_(*R,R*)DMEGNMe_2_(1,2)ch] (C6)

Colorless crystals, yield 62 % (0.145 g, 0.311 mmol).

^1^H NMR (400 MHz, CDCl_3_): δ = 3.62 (quint, *J* = 6.9 Hz, 2H, c), 3.53 (td, *J* = 10.2, 3.8 Hz, 1H, d), 3.27–3.20 (m, 2H, c`), 3.06 (s, 3H, b), 2.91 (s, 3H, b`), 2.72–2.65 (m, 1H, g), 2.57 (s, 3H, h), 2.30 (s, 3H, h`), 2.02–1.98 (m, 1H, e), 1.91–1.87 (m, 1H, e), 1.82–1.78 (m, 1H, f), 1.69–1.63 (m, 1H, f), 1.26–1.15 (m, 2H, f`), 1.14–1.02 (m, 2H, e`) ppm.

^13^C NMR (100 MHz, CDCl_3_): δ = 164.6 (a), 68.5 (g), 56.3 (d), 51.0 (c), 48.4 (c`), 44.9 (h), 38.1 (h`), 37.2 (b), 36.7 (b`), 32.6 (e), 24.9 (f), 24.6 (f`), 21.1 (e`) ppm.

HR-MS (ESI+, MeCN), m/z (%): calculated for [C_13_H_26_N_4_+H]^+^: 239.2230 (100); found: 239.2234 (100). No isotopic distribution of the complex or complex fragments were found.

IR (ATR, ṽ) = 3005 (vw), 2992 (vw), 2968 (w), 2940 (w), 2918 (w), 2893 (w), 2866 (w), 2849 (w), 2804 (vw), 1582 (vs), 1531 (w), 1504 (m), 1481 (w), 1470 (w), 1456 (m), 1441 (w), 1422 (s), 1406 (s), 1383 (w), 1364 (w), 1337 (w), 1315 (w), 1285 (m), 1269 (m), 1258 (w), 1221 (vw), 1196 (vw), 1182 (vw), 1173 (w), 1134 (vw), 1113 (vw), 1086 (w), 1042 (w), 1030 (m), 1018 (s), 957 (m), 943 (m), 907 (w), 876 (m), 849 (w), 829 (w), 800 (w), 781 (w), 737 (w), 660 (w), 602 (w), 588 (s), 532 (w), 490 (w), 476 (w), 442 (w) cm^–1^.

Elemental analysis calculated [%] for C_13_H_26_N_4_Br_2_Zn: C 33.68, H 5.65, N 12.09; found: C 33.75, H 5.71, N 12.00.

Information on the synthesis of the compound and original analysis data files are available via Chemotion Repository: 10.14272/reaction/SA-FUHFF-UHFFFADPSC-VJFBEMAPCX-UHFFFADPSC-NUHFF-LXMKZ-NUHFF-ZZZ

|  |  |
| --- | --- |

Figure S1: TGA measurement of **C1** at 150 °C (left) and 200 °C (right).

|  |  |
| --- | --- |

Figure S2: TGA measurement of **C4** at 150 °C (left) and 200 °C (right).

# 4. Crystallographic Data

| 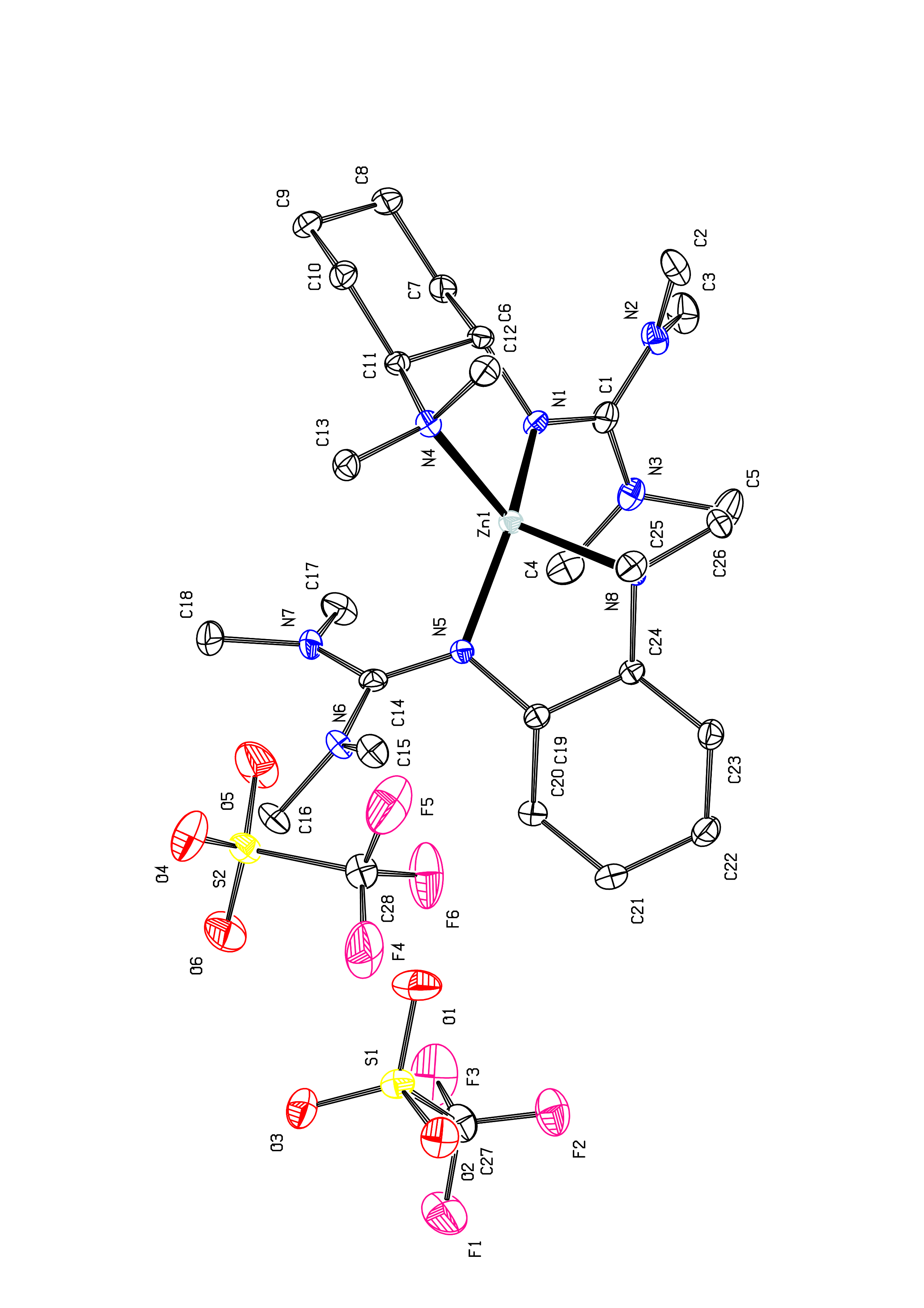  **C1** | 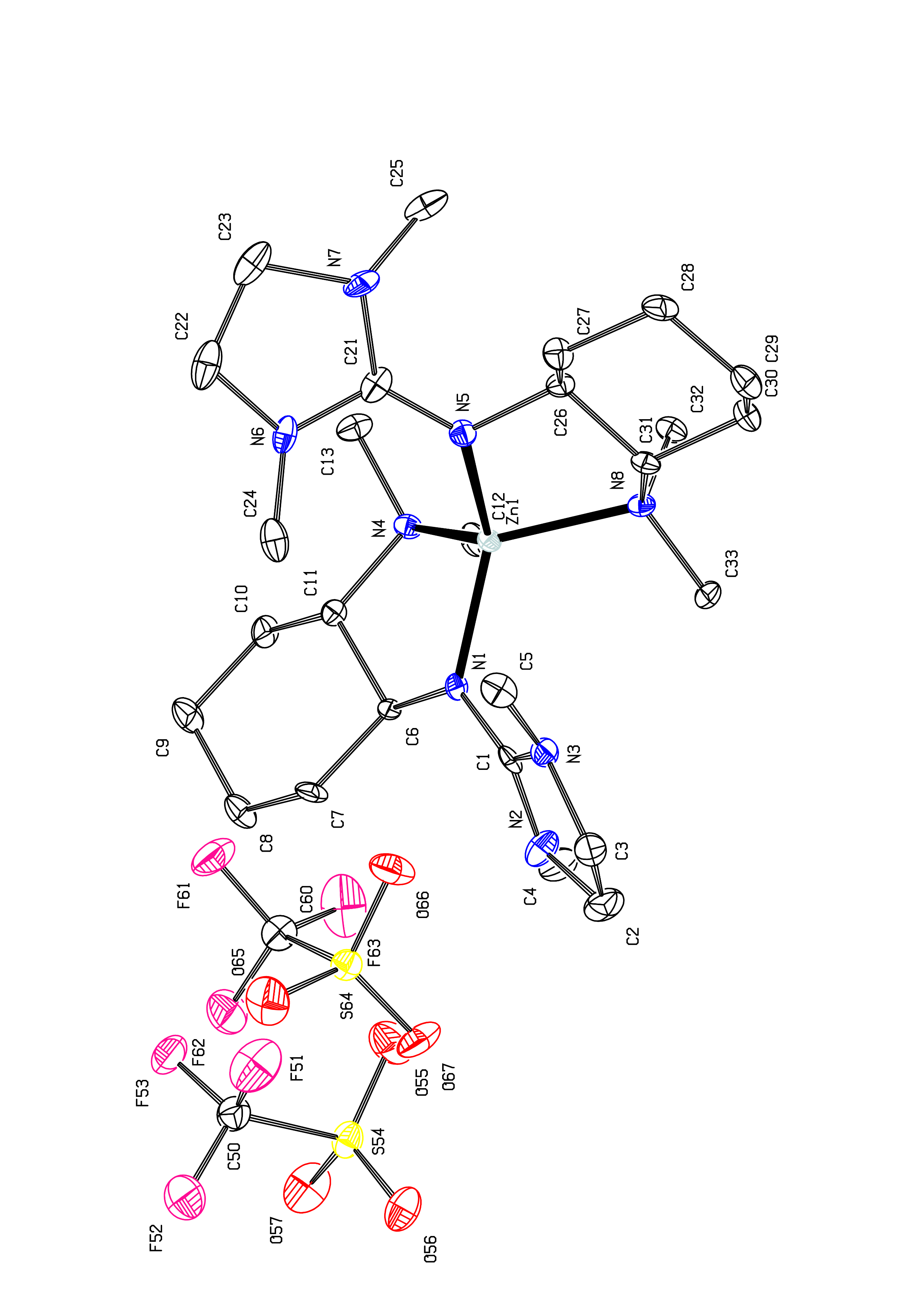  **C4** |
| --- | --- |

Figure S3: Molecular structures of **C1** (left) and **C4** (right) in the solid state (ellipsoids drawn at the 50% probability level). Hydrogen atoms are omitted for clarity.

Table S1: Crystallographic data and parameters of complex [Zn{(*R,R*)TMGNMe_2_(1,2)ch}_2_](OTf)_2_ (**C1**) and [Zn{(*R,R*)DMEGNMe_2_(1,2)ch}_2_](OTf)_2_ (**C4**).

| **parameter** | **C1** | **C4*** |
| --- | --- | --- |
| empirical formula  formula mass [g mol^−1^]  temperature [K]  wavelength [Å]  crystal system  space group  dimensions  a [Å]  b [Å]  c [Å]  *α* [°]  *β* [°]  *γ* [°]  V [Å^3^]  Z  *ρ*_cal_ [mg m^−3^]  µ [mm^−1^]  F(000)  crystal size [mm]  hkl range  reflections collected  independent reflections  R_int_  number of parameters  *goodness-of-fit* on F^2^  final R_1_ indices [I>2σ(I)]  wR_2_ indices (all data)  largest diff. peak, hole [e Å^−3^] | C_28_H_56_F_6_N_8_O_6_S_2_Zn  844.29  100  0.71073  monoclinic  *C*2/*c*  23.931(5)  16.656(3)  21.293(4)  90  116.00(3)  90  7628(3)  8  1.470  0.833  3552  0.130 × 0.100 × 0.060  -31<=h<=31  -22<=k<=22  -28<=l<=28  140492  9215  0.1157  472  0.846  0.0326  0.0674  0.669 / -0.532 | C_28_H_52_F_6_N_8_O_6_S_2_Zn  840.26  100  0.71073  monoclinic  *I*2/*a*  20.645(4)  16.655(3)  23.866(5)  90  115.10(3)  90  7431(3)  8  1.502  0.855  3520  0.160 × 0.090 × 0.020  -24<=h<=22  -20<=k<=20  -3<=l<=28  6892*  6892  *  469  1.115  0.0769  0.2223  4.722/ -1.747 |

* **C4** is a twin and the data set was treated with the TwinRotMat routine as implemented in platon (twofold rotation axis along [ 1 0 2] and twin law -1 0 0 0 -1 0 1 0 1 with BASF= 0.25383).^[13,14]^

| 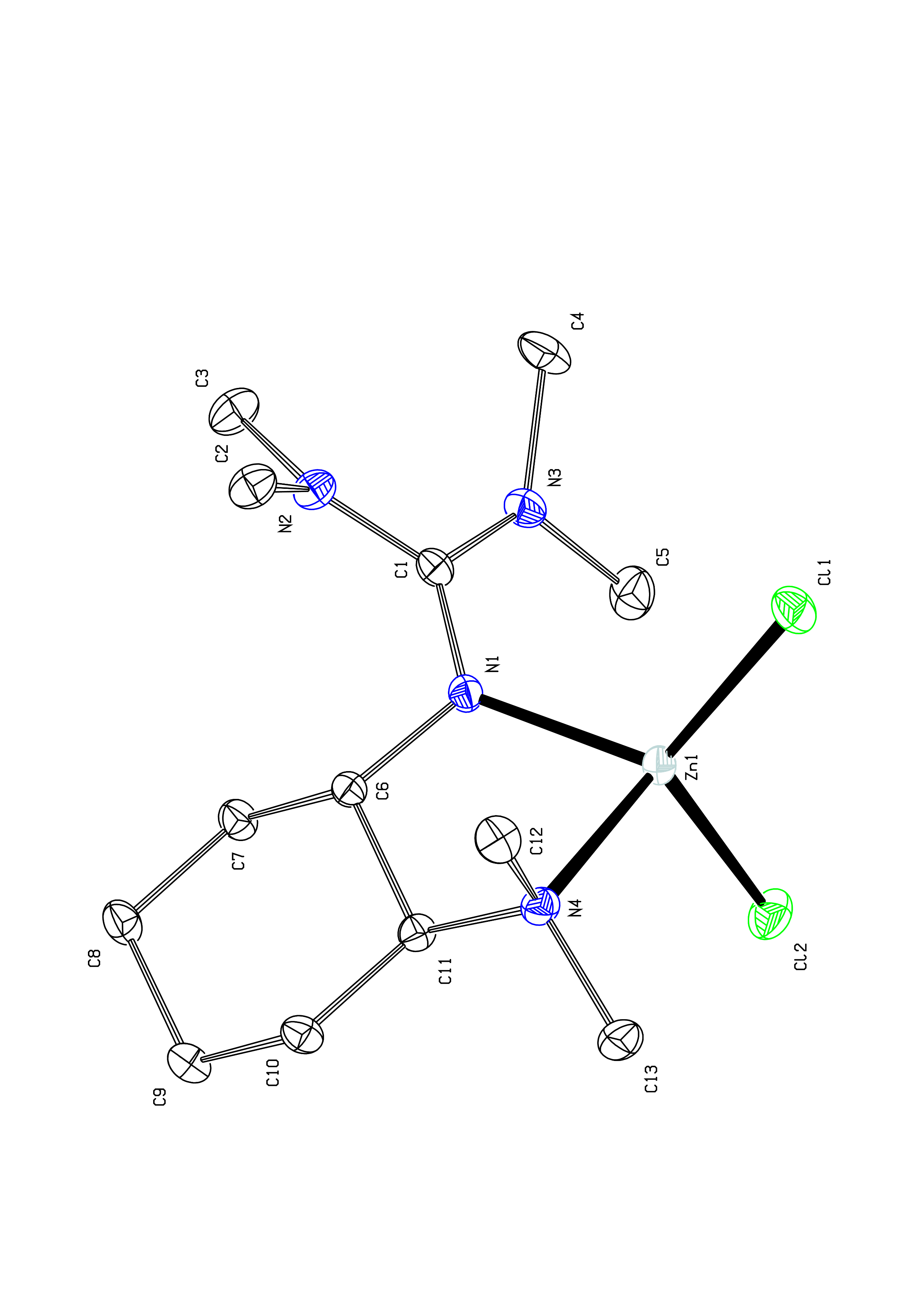  **C2** | 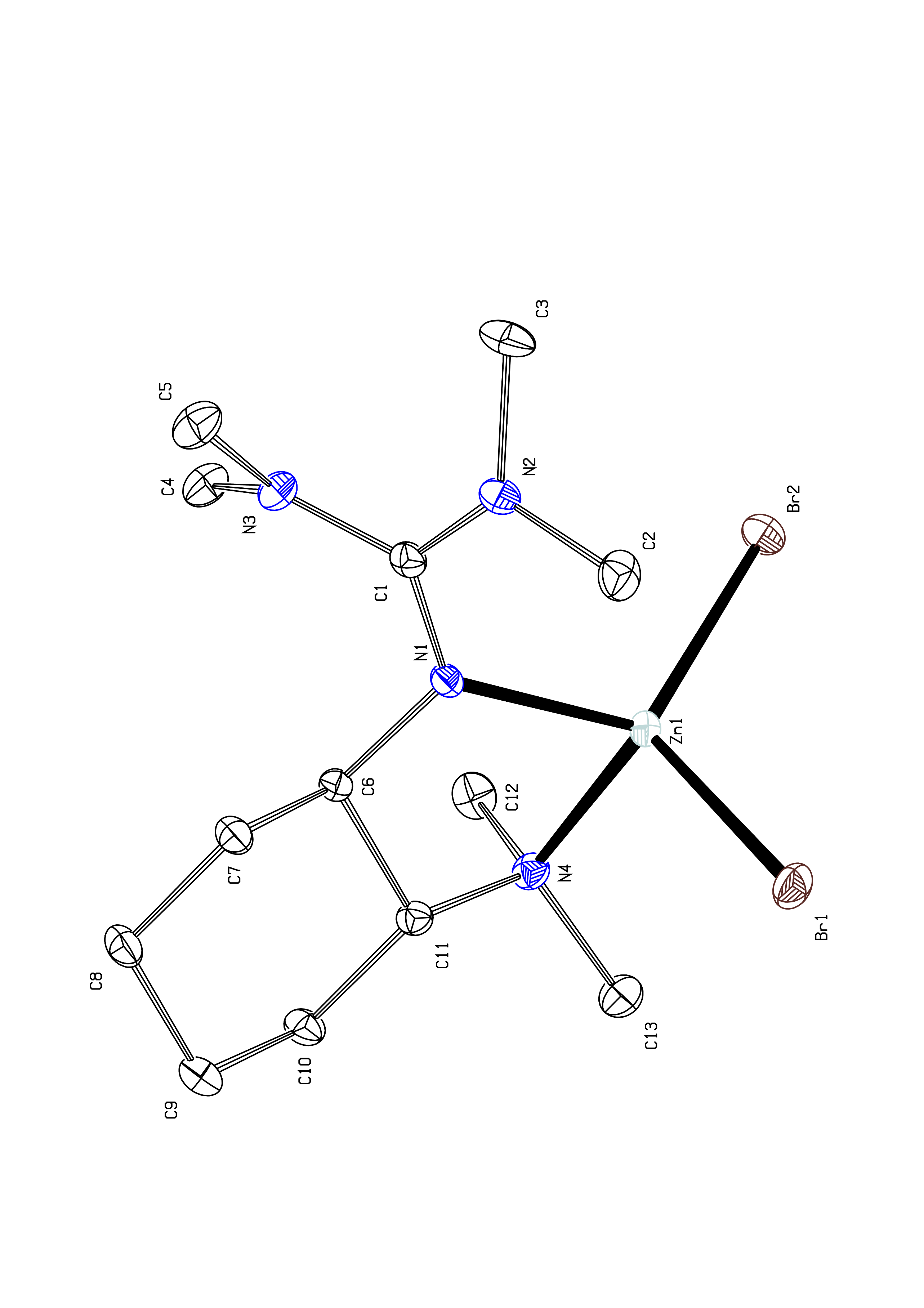  **C3** |
| --- | --- |

Figure S4: Molecular structures of **C2** (left) and **C4** (right) in the solid state (ellipsoids drawn at the 50% probability level). Hydrogen atoms are omitted for clarity.

Table S2: Crystallographic data and parameters of complex [ZnCl_2_(*R,R*)TMGNMe_2_(1,2)ch] (**C2**) and [ZnBr_2_(*R,R*)TMGNMe_2_(1,2)ch] (**C3**).

| **parameter** | **C2** | | **C3** |
| --- | --- | --- | --- |
| empirical formula  formula mass [g mol^−1^]  temperature [K]  wavelength [Å]  crystal system  space group  dimensions  a [Å]  b [Å]  c [Å]  *α* [°]  *β* [°]  *γ* [°]  V [Å^3^]  Z  *ρ*_cal_ [mg m^−3^]  µ [mm^−1^]  F(000)  crystal size [mm]  hkl range  reflections collected  independent reflections  R_int_  number of parameters  *goodness-of-fit* on F^2^  final R_1_ indices [I>2σ(I)]  wR_2_ indices (all data)  largest diff. peak, hole [e Å^−3^] | C_13_H_28_Cl_2_N_4_Zn  376.66  100  1.54186  monoclinic  *P*2_1_/*c*  11.042(2)  10.236(2)  15.890(3)  90  105.15(3)  90  1733.6(6)  4  1.443  4.764  792  0.210 × 0.140 × 0.090  -10<=h<=13  -12<=k<=9  -19<=l<=19  32058  3318  0.0220  187  1.067  0.0232  0.0625  0.329/ -0.411 | | C_13_H_28_Br_2_N_4_Zn  465.58  100  1.54186  monoclinic  *P*2_1_/*c*  11.237(2)  10.381(2)  16.093(3)  90  105.91(3)  90  1805.3(7)  4  1.713  7.039  936  0.200 × 0.150 × 0.080  -10<=h<=13  -12<=k<=12  -19<=l<=18  34343  3408  0.0152  187  1.204  0.0190  0.0485  0.324 / -0.586 |
| 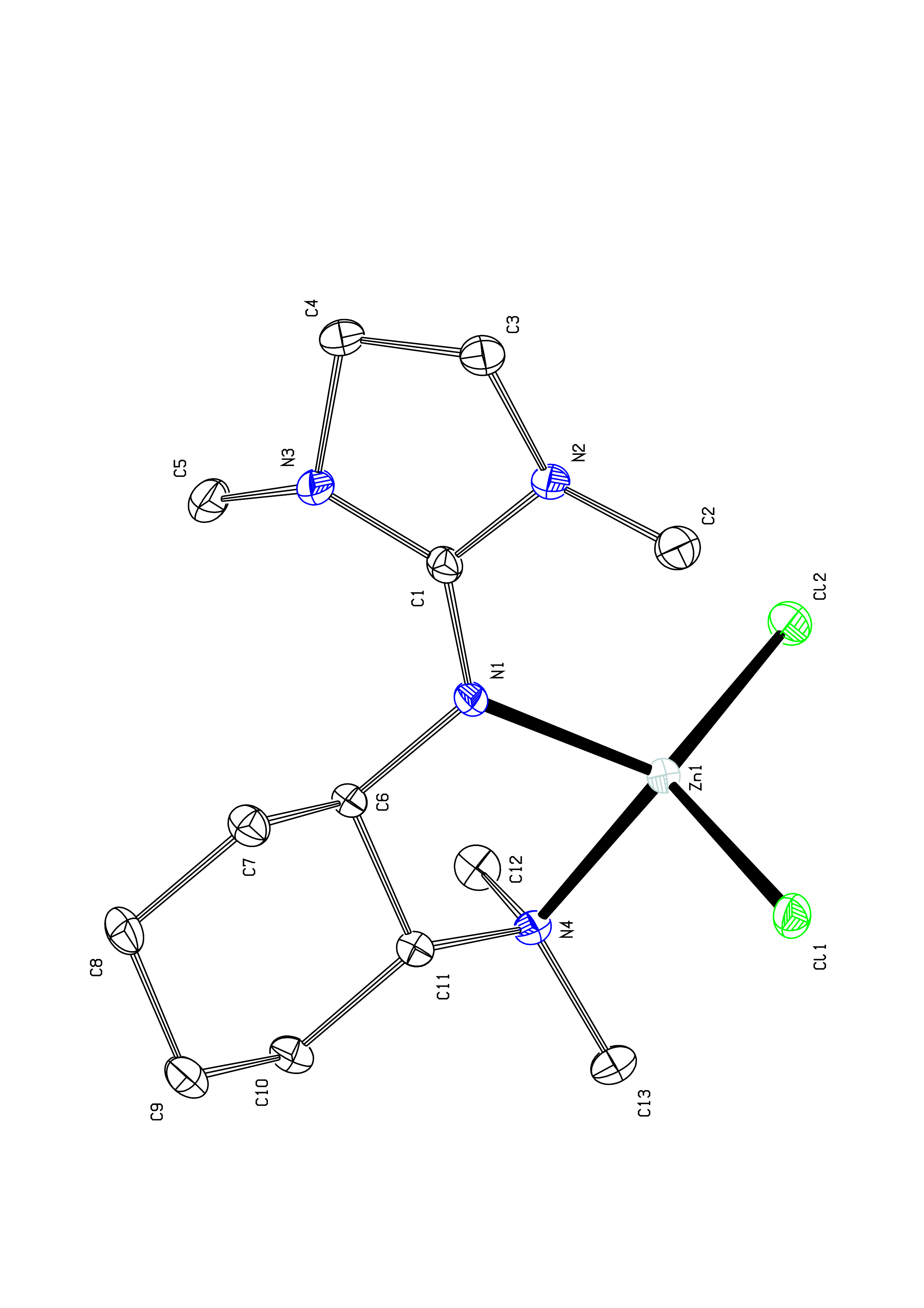  **C5** | | 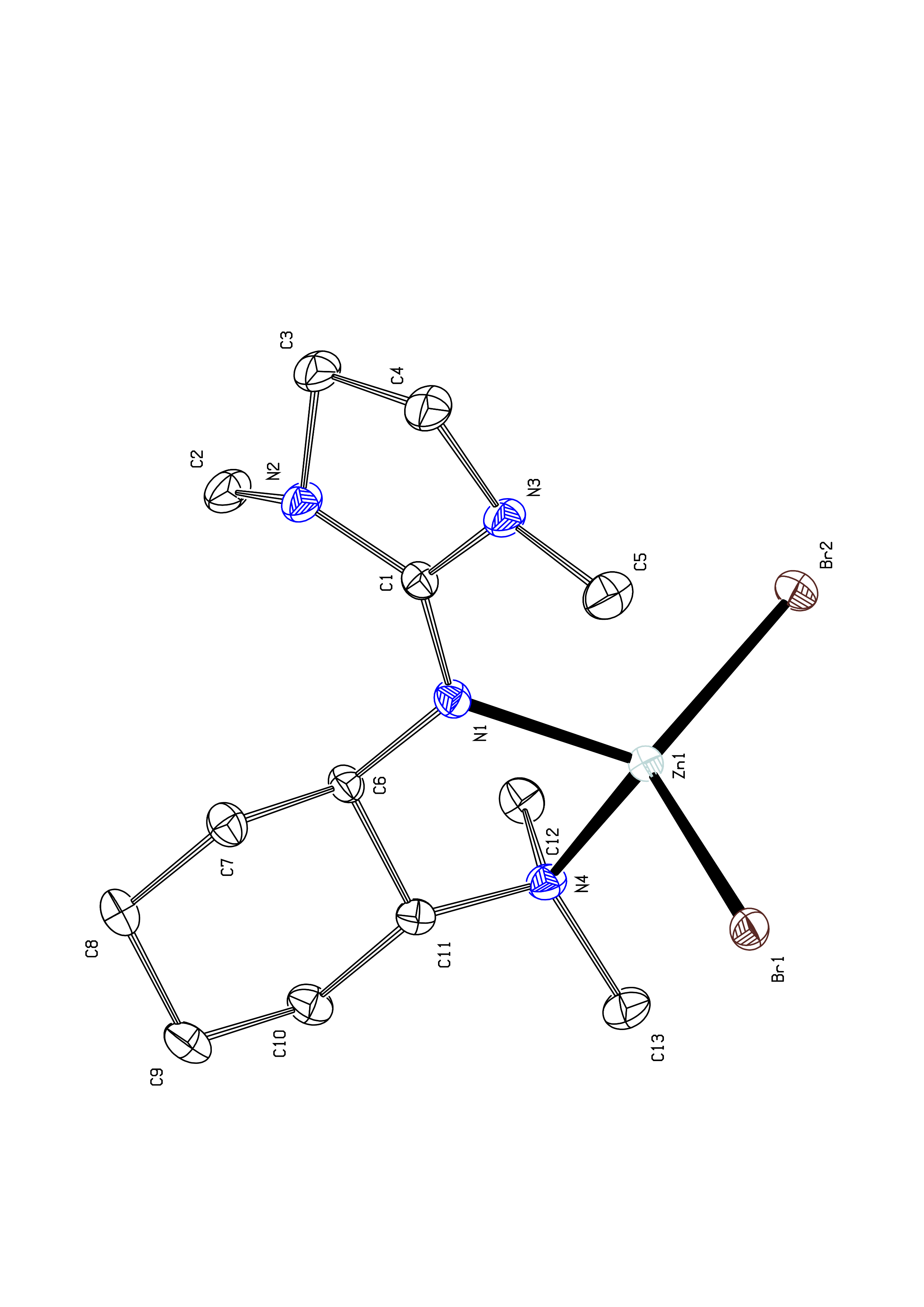  **C6** | |

Figure S5: Molecular structures of **C5** (left) and **C6** (right) in the solid state (ellipsoids drawn at the 50% probability level). Hydrogen atoms are omitted for clarity.

Table S3: Crystallographic data and parameters of complex [ZnCl_2_(*R,R*)DMEGNMe_2_(1,2)ch] (**C5**) and [ZnBr_2_(*R,R*)DMEGNMe_2_(1,2)ch] (**C6**).

| **parameter** | **C5** | **C6** |
| --- | --- | --- |
| empirical formula  formula mass [g mol^−1^]  temperature [K]  wavelength [Å]  crystal system  space group  dimensions  a [Å]  b [Å]  c [Å]  *α* [°]  *β* [°]  *γ* [°]  V [Å^3^]  Z  *ρ*_cal_ [mg m^−3^]  µ [mm^−1^]  F(000)  crystal size [mm]  hkl range  reflections collected  independent reflections  R_int_  number of parameters  *goodness-of-fit* on F^2^  final R_1_ indices [I>2σ(I)]  wR_2_ indices (all data)  largest diff. peak, hole [e Å^−3^] | C_13_H_26_Cl_2_N_4_Zn  374.65  100  0.71073  monoclinic  *P*2_1_/*c*  10.823(2)  9.895(2)  16.483(3)  90  103.70(3)  90  1715.0(6)  4  1.451  1.740  784  0.240 × 0.190 × 0.120  -16 <= h <= 16  -13 <= k <= 14  -24 <= l <= 15  5786  66958  0.0386  185  1.050  0.0243  0.0598  0.382 / -0.498 | C_13_H_26_Br_2_N_4_Zn  463.57  100  1.54186  monoclinic  *P*2_1_/*c*  11.145(2)  9.902(2)  16.721(3)  90  103.97(3)  90  1790.8(7)  4  1.719  7.096  928  0.200 × 0.140 × 0.100  -13<=h<=10  -10<=k<=12  -20<=l<=19  34620  3437  0.0162  185  1.086  0.0180  0.0443  0.327/ -0.410 |

Table S4: Comparison of selected bond lengths, angles and structure parameters of **C1** with literature known complexes.

| complex | Zn–N_gua_ [Å] | Zn–N_amine_ [Å] | N–Zn–N [°] | *ρ* | *τ*_4_ |
| --- | --- | --- | --- | --- | --- |
| [Zn{(*R,R*)TMGNMe_2_(1,2)ch}_2_](OTf)_2_ (C1) | 1.9939(15) 1.9829(16) | 2.0775(16) 2.0930(15) | 86.8(6)/ 86.4(6)^[c]^ | 0.99  0.98 | 0.69 |
| [Zn(TMGhydroqu)_2_](OTf)_2_^[43]^ | 1.959(3) 1.950(3) | 2.034(3) 2.042(3) | 84.5(1)/ 84.6(1)^[c]^ | 0.99 0.99 | 0.71 |
| [Zn{(R,R)DMEG_2_(1,2)ch}_2_](OTf)2·THF^[44]^ | 1.991(6), 2.067(8), 2.008(7), 2.004(7) | – | 86.2(3)/ 86.5(3)^[c]^ | 0.95  0.96 | 0.62 |
| [a] *ρ*=2a/(b+c).^[45]^[b] *τ*_4_*=*[360°−(α+β)]/141°, with *τ*_4_=0 indicating square-planar coordination and *τ*_4_=1 for tetrahedral coordination.^[46]^ [c] Two chelate angles of both ligands to the Zn atom are reported. | | | | | |

# 5. Polymerization Details

| 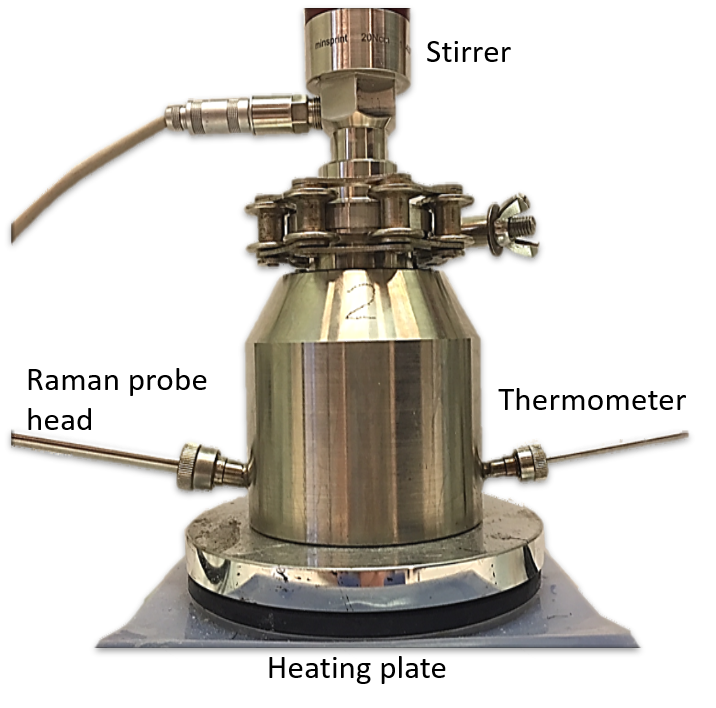 | 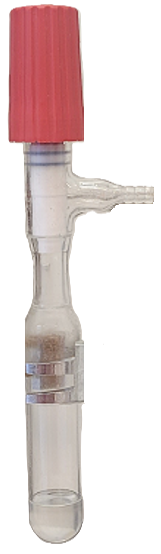 |
| --- | --- |

Figure S6: Construction of the Raman reactor (left) and Young-type Schlenk tube (right).

**Polymerization under bulk conditions in a steal reactor monitored by in situ Raman spectroscopy:** The reactor was heated at 150 °C under vacuum and flushed with argon (3 ×). The reactions were conducted under argon atmosphere at a stirring speed of 260 rpm. After adding the reaction mixture and closing the reactor, detection started using in situ Raman spectroscopy, which marks the start of a ROP experiment (*t* = 0). In a nitrogen filled glovebox, recrystallized *L*-lactide (8.00 g, 55.5 mmol) and the catalyst were weighed in according to the specific [M]/[I]-ratio, mixed in an agar mortar and the mixture were transferred into a glass vial. If co-initiator was used, benzyl alcohol (BnOH) were added with an Eppendorf pipette. Or the catalyst was weighed in a glass vial according to the respective [M]/[I]-ratio and distilled ε-caprolactone (8.00 mL, 8.64 g, 75.7 mmol, 1.08 g cm^-1^) was added. If co-initiator was used, BnOH were added with an Eppendorf pipette. The exact weight portions are listed in Table S5. The glass vial was removed from the glovebox and the reaction mixture was transferred into the reactor in argon counterflow. After the reaction was completed, the polymer was removed from the reactor and a sample was taken to determine the conversion via^1^H NMR spectroscopy. The crude polymer was dissolved in an appropriate amount of DCM, then precipitated in ethanol at room temperature and purified under vacuum. A sample was taken for SEC measurements to determine the molar mass and dispersity.

**Polymerization under solvent conditions in Schlenk tubes:** In a nitrogen filled glovebox, recrystallized *L*-lactide (1.153 g, 8.00 mmol) and the catalyst were weighed in according to the specific [M]/[I]-ratio and mixed in an agar mortar. The exact weight portions are listed in Table S5. The reaction mixture was transferred into the Young-type Schlenk tube and removed from the glovebox. Toluene (8 mL) was added to the Schlenk tube under inert gas atmosphere. After fixing the Schlenk tube in a preheated oil bath 150 °C and stirred with a magnetic stirrer at 260 rpm, the reaction time started (*t* = 0). The reaction was stopped by removing the Schlenk tube from the oil bath and taking it under cold running water. After the reaction was completed, a sample was taken for ^1^H NMR spectroscopy to determine the conversion. The polymer was dissolved in an appropriate amount of DCM, then precipitated in ethanol at room temperature and purified under vacuum. A sample was taken for SEC measurements to determine the molar mass.

Table S5. Exact weight portions for polymerization with **C1** of recrystallized L-lactide (8 g) in bulk at 150 °C,^[a]^ recrystallized *L*-lactide in toluene (8.0 mL) at 100°C,^[b]^ and ε-caprolactone (8 mL) in bulk at 150 °C^[c]^ using a stirrer speed of 260 rpm.

| # | [M]:[C1]:[I] | [M] | m(C1) [mg] | V(BnOH) [μL] |
| --- | --- | --- | --- | --- |
| 1^[a]^ | 500:1 | *L*-lactide | 93.7 | – |
| 2^[a]^ | 950:1 | *L*-lactide | 49.3 | – |
| 3^[a]^ | 1000:1 | *L*-lactide | 46.9 | – |
| 4^[a]^ | 1460:1 | *L*-lactide | 32.1 | – |
| 5^[a]^ | 1500:1 | *L*-lactide | 31.2 | – |
| 6^[a]^ | 1500:1:1 | *L*-lactide | 31.2 | 3.8 |
| 7^[a]^ | 1915:1 | *L*-lactide | 24.5 | – |
| 8^[a]^ | 2000:1 | *L*-lactide | 23.3 | – |
| 9^[a]^ | 2500:1 | *L*-lactide | 18.7 | – |
| 10^[a]^ | 2500:1:1 | *L*-lactide | 18.7 | 2.3 |
| 11^[b]^ | 500:1 | *L*-lactide | 13.5 | – |
| 12^[b]^ | 600:1 | *L*-lactide | 11.3 | – |
| 13^[b]^ | 650:1 | *L*-lactide | 10.4 | – |
| 14^[b]^ | 750:1 | *L*-lactide | 9.0 | – |
| 15^[b]^ | 800:1 | *L*-lactide | 8.4 | – |
| 16^[c]^ | 1000:1 | ε-caprolactone | 63.9 | – |
| 17^[c]^ | 1250:1 | ε-caprolactone | 51.1 | – |
| 18^[c]^ | 1500:1 | ε-caprolactone | 42.6 | – |
| 19^[c]^ | 1500:1:1 | ε-caprolactone | 42.6 | 5.2 |
| 20^[c]^ | 1500:1.10 | ε-caprolactone | 42.6 | 52.5 |
| 21^[c]^ | 2000:1 | ε-caprolactone | 32.0 | – |
| 22^[c]^ | 2500:1 | ε-caprolactone | 25.6 | – |

PLA

**methyl groups**

l-lactide

*meso*-lactide

PLA

l-lactide

**methine groups**

Figure S7: Exemplary ^1^H NMR spectrum (400 MHz, CDCl_3_) of recrystallized *L*-lactide polymerization with **C1** (150 °C, 260 rpm, [M]/[I] = 1000:1, 93% conversion).

Figure S8: Exemplary DSC thermogram of cooling and second heating phase of PLA (black lines) made of l-lactide (M]/[I] ratio 1000:1, 150 °C, 260 rpm) and PCL (red lines) made of ε-caprolactone (M]/[I] ratio 1000:1, 150 °C, 260 rpm) using the catalyst **C1**. Tm of PLA = 176 °C and PCL = 58 °C.

Figure S9: MALDI-ToF-MS results for PLA, sample produced by catalyst **C1** and co-initiator BnOH with [M]/[I]/[Co-I] ratio 1000:1:10. Exemplary calculations: 15115.42 = 182 × (72.02 g mol^−1^) + (ligand + Zn) + Na^+^.

Table S6: End groups determined by MALDI-ToF-MS for PLA with complex **C1**.

| End groups | **C1** |
| --- | --- |
| OH  EtOH  BnOH  Ligand  1× ligand + Zn  Complex  Complex - OTf | Yes  Yes  Yes  Yes  Yes  Yes  Yes |

Conditions: [M]:[**C1**]:[BnOH] ratio 1000:1:10, 150 °C, 5 min, 260 rpm, benzyl alcohol as co-initiator; **C1**: conversion 91 %.

EtOH and OH end groups are present due to the purification of the polymer.

Figure S10: Exemplary plot of SEC analysis of PLA (150 °C, [M]/[I] ratio of 1000:1, purified in EtOH).

## 5.1 l-Lactide polymerization in bulk


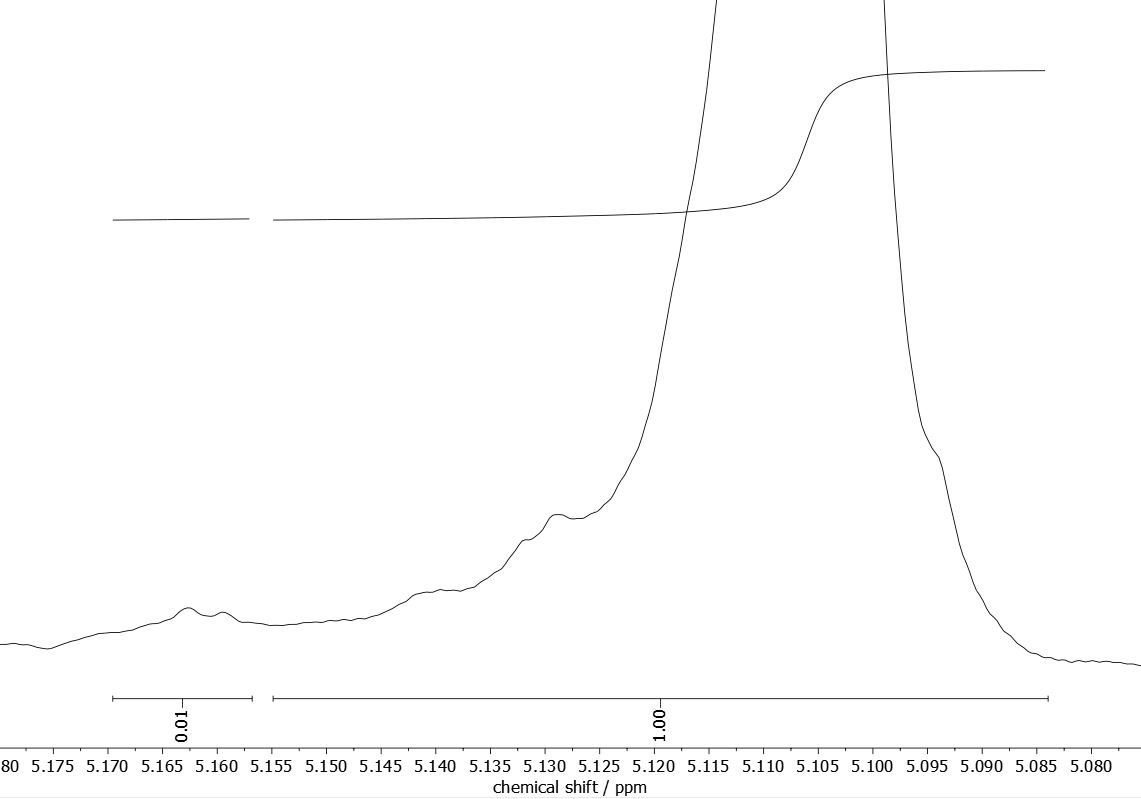
Figure S11: Extract from a homonuclear decoupled ^1^H NMR spectrum from the polymerization of *L*-lactide at 150 °C with **C1**, [M]/[I] ratio of 1000:1 with i = isotactic and s = syndiotactic; *P*_r_ value of 0.14. Calculated from the equation: $P_{r}= \sqrt{2\times\left[ sis \right]}$*, P*_r_ + *P*_m_ = 1.^[47]^

sii

sis

iis

isi

iii

Table S7: Polymerization of recrystallized l-lactide in bulk using [Zn(TMGNMe_2_ch)_2_](OTf)_2_ (**C1**).^[a]^

| **#** | **[M]/[I]** | ***k*_obs_ · 10^-2 [d]^**  **[s^-1^]** | ***t***  **[s]** | ***X*_NMR_^[e]^**  **[%]** | ***M*_n,exp_ ^[f]^**  **[g mol^-1^]** | ***M*_n,theo_ ^[g^**  **[g mol^-1^]** | ***Ð* ^[h]^** |
| --- | --- | --- | --- | --- | --- | --- | --- |
| **1** | 500:1 | 11.1±0.6 | 162 | 93 | 80 200 | 67 000 | 1.5 |
| **2** | 950:1 | 5.0 | 105 | 94 | 76 300 | 128 700 | 1.5 |
| **3** | 1000:1 | 5.1±0.5 | 75 | 94 | 57 300 | 135 500 | 1.6 |
| **4^[b]^** | 1000:1 | 4.5 | 105 | 67 | 60 300 | 96 600 | 1.8 |
| **5** | 1460:1 | 2.8 | 165 | 82 | 66 700 | 172 600 | 1.6 |
| **6** | 1500:1 | 3.0 | 75 | 84 | 59 400 | 162 100 | 1.7 |
| **7** | 1680:1 | 2.4 | 162 | 65 | 49 000 | 157 400 | 1.7 |
| **8** | 1915:1 | 1.4 | 165 | 56 | 78 100 | 154 600 | 1.4 |
| **9** | 2000:1 | 1.5 | 120 | 50 | 36 000 | 144 100 | 1.9 |
| **10** | 2500:1 | 0.6±0.05 | 165 | 26 | 33 000 | 93 700 | 1.6 |
| **11^[c]^** | 1500:1:1 | 2.7 | 75 | 79 | 118 000 | 85 400 | 1.6 |
| **12^[c]^** | 1500:1:10 | 5.8 | 75 | 96 | 12 800 | 18 900 | 1.9 |
| [a] Reaction conditions: reactor, Ar atmosphere, recrystallized l-lactide (8.0 g, 55.5 mmol), **C1** (depending on the [M]/[I] ratio), 150 °C, 260 rpm. [b] Technical l-lactide. [c] Benzyl alcohol as coinitiator. [d] Observable rate constant *k*_obs_ determined by plotting $\ln\left( \frac{\left[ \mathrm{LA} \right]_{0}}{\left[ \mathrm{LA} \right]_{t}} \right)$*vs*. t. [e] Determined by ^1^H-NMR spectroscopy. [f] Determined *via* SEC analysis in THF. [g] Calculated using the following equation: Molar mass x conversion x monomer ratio. [h] Calculated using the following equation: *M*_w_ / *M*_n_. | | | | | | | |

Figure S12: Semi-logarithmic plots for the polymerization of recrystallized l-lactide catalyzed by **C1**.

## 5.2 l-Lactide polymerization in solution

Table S8: Polymerization of recrystallized l-lactide in solution using [Zn(TMGNMe_2_ch)_2_](OTf)_2_ (**C1**).^[a]^

| **#** | **[M]/[I]** | ***k*_obs_ · 10^-3 [b]^**  **[s^-1^]** | ***t***  **[s]** | ***X*_NMR_^[c]^**  **[%]** | ***M*_n,exp_ ^[d]^**  **[g mol^-1^]** | ***M*_n,theo_ ^[e]^**  **[g mol^-1^]** | ***Ð* ^[f]^** |
| --- | --- | --- | --- | --- | --- | --- | --- |
| **1** | 500:1 | 4.7±0.0 | 420 | 59 | 69 200 | 42 500 | 1.9 |
| **2** | 600:1 | 3.7 | 540 | 59 | 81 400 | 51 000 | 1.5 |
| **3** | 650:1 | 2.8 | 660 | 43 | 34 100 | 40 300 | 2.2 |
| **4** | 750:1 | 2.0 | 1020 | 74 | 56 000 | 78 000 | 2.1 |
| **5** | 800:1 | 2.3±0.3 | 840 | 66 | 66 600 | 76 100 | 1.7 |
| [a] Reaction conditions: Schlenk tube, N_2_ atmosphere, recrystallized l-LA (1.153 g, 0.008 mol), **C1** (depending on the [M]/[I] ratio), toluene (8 mL), 100 °C, 260 rpm. [b] Observable rate constant *k*_obs_ determined by plotting $\ln\left( \frac{\left[ \mathrm{LA} \right]_{0}}{\left[ \mathrm{LA} \right]_{t}} \right)$*vs*. t. [c] Determined by ^1^H NMR spectroscopy. [d] Determined *via* SEC analysis in THF. [e] Calculated using the following equation: Molar mass x conversion x monomer ratio. [f] Calculated using the following equation: *M*_w_ / *M*_n_. | | | | | | | |

Figure S13: Semi-logarithmic plots for the polymerization of recrystallized l-lactide in toluene catalyzed by **C1**.

Figure S14: Plot of *k*_obs_ against catalyst concentration with recrystallized l-lactide for the determination of the propagation rate constant *k*_p_. [M]/[I]=500:1 to 800:1. The polymerization straight line is not a straight line of origin due to a small extent of catalyst deactivation.

## 5.3 ε-Caprolactone polymerization

PCL

ε-caprolactone

Figure S15: Exemplary ^1^H NMR spectrum (400 MHz, CDCl_3_) of polymerization with distilled ε-caprolactone and **C1** (150 °C, 260 rpm, [M]/[I] = 2000:1, 47% conversion).

Figure S16: Exemplary plot of SEC analysis of PCL (150 °C, [M]/[I] ratio of 1000:1, purified in EtOH).

Table S9: Polymerization of distilled ε-caprolactone in bulk using [Zn(TMGNMe_2_ch)_2_](OTf)_2_ (**C1**).^[a]^

| # | [M]/[I] | *k_obs_* · 10^-2 [c]^  [s^-1^] | *t*  [s] | *X_NMR_* ^[d]^  [%] | *M_n,exp_* ^[e]^  [g mol^-1^] | *M_n,theo_* ^[f]^  [g mol^-1^] | *Ð* ^[g]^ |
| --- | --- | --- | --- | --- | --- | --- | --- |
| 1 | 1000:1 | 3.9±0.02 | 75 | 100 | 127 000 | 114 000 | 2.0 |
| 2 | 1250:1 | 2.4 | 75 | 72 | 100 000 | 103 000 | 1.8 |
| 3 | 1500:1 | 1.4 | 75 | 71 | 59 500 | 122 000 | 1.8 |
| 4 | 2000:1 | 1.1 | 105 | 47 | 53 000 | 107 000 | 1.6 |
| 5 | 2500:1 | 0.4±0.04 | 165 | 31 | 40 600 | 88 500 | 1.7 |
| 6^[b]^ | 1500:1:1 | 3.3±0.28 | 102 | 97 | 84 300 | 83 000 | 1.8 |
| 7^[b]^ | 1500:1:10 | 6.4±0.01 | 165 | 99 | 25 900 | 15 400 | 1.7 |
| [a] Reaction conditions: reactor, Ar atmosphere, distilled ε-CL (8.0 mL, 75.7 mmol), C1 (depending on the [M]/[I] ratio), 150 °C, 260 rpm. [b] Benzyl alcohol as coinitiator. [c] Observable rate constant determined by plotting $\mathbf{ln}\left( \frac{\left[ \mathbf{LA} \right]_{\mathbf{0}}}{\left[ \mathbf{LA} \right]_{\boldsymbol{t}}} \right)$*vs*. t. [d] Determined by ^1^H NMR spectroscopy. [e] Determined *via* SEC analysis in THF. [f] Calculated using the following equation: Molar mass x conversion x monomer ratio. [g] Calculated using the following equation: *M*_w_ / *M*_n_. | | | | | | | |

Figure S17: Semi-logarithmic plots for the polymerization of distilled ε-caprolactone catalyzed by **C1**.

# 6. Chemical recycling

**Polyester alcoholysis in solution:** In a nitrogen filled glovebox, **C1** (1.0–2.0 mol%, regarding the ester bonds) and THF (4 mL) were weighed into a Young-type Schlenk tube. The respective polymer (3.47 mmol, 1.0 eq, bio-mi Ltd.) was added in nitrogen counterflow and dissolved with an external heat source. The exact weight portions are listed in Table S9. The Young-type Schlenk tube was placed in an oil bath preheated to 60 °C and stirred with a magnetic stirrer at 260 rpm. To start the depolymerization, the respective alcohol (24.3 mmol, 7.1 eq) was added under nitrogen counterflow. Samples were taken after defined time intervals, and the progress of the reaction was monitored by ^1^H NMR spectroscopy in CDCl_3_ according to literature.

**Polyester alcoholysis under solvent-free conditions:** In a nitrogen filled glovebox, **C1** (1.0–2.0 mol %, regarding the ester bonds) was weighed into a Young-type Schlenk tube. The respective polymer (3.47 mmol, 1.0 eq, bio-mi Ltd.) was added in nitrogen counterflow and dissolved with an external heat source. The Young-type Schlenk tube was placed in an oil bath preheated to 150 °C and stirred with a magnetic stirrer at 260 rpm. To start the depolymerization, the respective alcohol (24.3 mmol, 7.1 eq) was added under nitrogen counterflow. Samples were taken after defined time intervals, and the progress of the reaction was monitored by ^1^H NMR spectroscopy in CDCl_3_.

**Closed-loop recycling:** The recycling of PLA to LA was carried out following the synthesis procedure of Williams et al.^[48]^ In a glovebox filled with nitrogen, **C1** (29.3 mg, 0.0347 mmol, 0.01 eq) was added to a Young-type Schlenk tube or sublimation apparatus. PLA (250 mg, 3.47 mmol, 1.0 eq) was added using nitrogen countercurrent. The reaction vessel was placed in an oil bath preheated to 150 °C under reduced pressure. The reaction was stopped under running tap water after 24 h or the obtained lactide was separated from the reaction residue.

**Catalyst recycling:** The product mixture from depolymerization under solvent free conditions with methanol or ethanol at 150 °C was used for catalyst recycling. The liquids were removed under reduced pressure. PLA (250 mg, 24.3 mmol, 1.0 eq) was added to the oily residue. The reaction was started by injecting methanol (1.0 mL, 24.3 mmol, 7.1 eq) or ethanol (1.4 mL, 24.3 mmol, 7.1 eq) and subsequently fixing the Young-type Schlenk tube in an oil bath preheated to 150°C.. After 1 h, the reaction was stopped, and another recycling run was started as described above.

**Recycling of polyethylene terephthalate (PET) to dimethyl terephthalate (DMT) or bis (hydroxyethyl) terephthalate (BHET):** The reaction was conducted according to the literature-known process of Jones *et al*.^[49]^ In a nitrogen-filled glovebox, **C1** (27.3 mg, 0.026 mmol, 0.01 eq) was added to a Young-type Schlenk tube. PET (250 mg, 2.60 mmol, 1.0 eq, regarding the ester bonds, bio-mi Ltd.) was added and methanol or ethylene glycol (2 mL) were added using nitrogen counterflow. The Schlenk tube was fixed in an oil bath preheated to 150 or 180 °C indicating the start of the depolymerization (*t* = 0). After 3 or 4 h, the reaction was stopped under running tap water. Deionized water (10 mL) was added to the reaction mixture and the Schlenk tube was stored at 4 °C to precipitate the product as colorless crystals. After a few days, the product was washed with water, filtered, and dried under vacuum (DMT: *Y*=55 % and BHET: *Y*=76 %).

Table S10: Exact weighed amounts of polyester, alcohol, catalyst, THF and temperature used for the chemical recycling experiments.

| **#** | **T** | | **m_C1_** | | **m_PLA_** | **m_PET_** | **m_PCL_** | **m_PLA/PCL blend_** | **THF** | **alcohol** |
| --- | --- | --- | --- | --- | --- | --- | --- | --- | --- | --- |
|  | **[°C]** | | **[mg]** | | **[mg]** | **[mg]** | **[mg]** | **[mg]** | **[mL]** | **[mL]** |
| **MeOH** | |  | |  |  |  |  |  |  |  |
| 1  2  3  4  5  6  7  8  9 | 60  60  60  60  60  150  150  150  150 | | 29.3  36.6  44.0  51.3  58.6  29.3  29.3  29.3 | | 250  250  250  250  250  250  –  –  – | –  –  –  –  –  –  250  –  – | –  –  –  –  –  –  –  396  – | –  –  –  –  –  –  –  –  250 | 4  4  4  4  4  –  –  –  – | 1  1  1  1  1  1  2  1  1 |
| **EtOH** | |  |  |  |  |  |  |  |  |  |
| 10  11  12  13  14 | 60  150  150  150  150 | | 29.3  29.3  29.3  29.3  29.3 | | 250  250  –  –  – | –  –  –  –  – | –  –  –  396  – | –  –  –  –  250 | 4  –  –  –  – | 1.4  1.4  1.4  1.4  1.4 |
| ***n*-BuOH** | |  |  |  |  |  |  |  |  |  |
| 15 | 150 | | 29.3 | | 250 | – | – | – | – | 2.2 |
| ***i*-BuOH** | |  |  |  |  |  |  |  |  |  |
| 16 | 150 | | 29.3 | | 250 | – | – | – | – | 2.3 |
| **BnOH** | |  |  |  |  |  |  |  |  |  |
| 17 | 150 | | 29.3 | | 250 | – | – | – | – | 2.5 |
| ***t*-BuOH** | |  |  |  |  |  |  |  |  |  |
| 18 | 150 | | 29.3 | | 250 | – | – | – | – | 2.3 |
| **Ethylene glycol** | | | |  |  |  |  |  |  |  |
| 19 | 180 | | 29.3 | | – | 250 | – | – | – | 2 |

*

*

*

*

Figure S18: Exemplary ^1^H NMR spectrum (400 MHz, CDCl_3_) of PLA (250 mg) methanolysis (7.1 eq MeOH) in THF with 1 mol% **C1** (regarding the polymer ester bonds). This is representative for the alcoholysis of PLA. Black and blue labeling: internal methine group of the polymer and methine group of the Ω-chain end (5.15 ppm), red labeling: methine group of the α-oligomers (4.33 ppm) and green labeling: methine group of the lactate and for ethyllactate the CH_2_ group (4.25 ppm) according to literature.^[49–52]^

The data obtained for PLA degradation with MeOH, EtOH, *n*-BuOH, *i*-BuOH and BnOH were analyzed according to literature.^[50–52]^

Figure S19: Semilogarithmic plots of ln(PLA_0_/PLA_t_) against time for the methanolysis of PLA (250 mg) for different **C1** loadings (1.0 mol%–2.0 mol%, regarding the polymer ester bonds) at 60 °C in THF (4 mL).

Table S11: Results of polyester alcoholysis.^[a]^

| # | Polyester | alcohol | *c* (C1)  [mol%] | T  [°C] | *t*  [min] | *X*_Polyester_  [%]^[d]^ | *S*_Product_  [%]^[d]^ | *Y*_Product_  [%]^[d]^ | *k_obs·_*10^-3^  [min^-1^] ^[e]^ |
| --- | --- | --- | --- | --- | --- | --- | --- | --- | --- |
| 1 | PLA | MeOH | 1.00 | 60 | 360  1440  2880 | 52  75  82 | 41  60  67 | 22  45  54 | 2.3±0.3 |
| 2 | PLA | MeOH | 1.25 | 60 | 360 | 58 | 52 | 30 | 2.7 |
| 3 | PLA | MeOH | 1.50 | 60 | 360 | 80 | 65 | 52 | 4.3 |
| 4 | PLA | MeOH | 1.75 | 60 | 360 | 85 | 68 | 58 | 5.3 |
| 5 | PLA | MeOH | 2.00 | 60 | 360 | 92 | 75 | 68 | 7.3±0.0 |
| 6^[b]^ | PLA | MeOH | 1.00 | 150 | 15  30 | 100  100 | 94  94 | 94  94 | –  – |
| 7 | PLA | EtOH | 1.00 | 60 | 360  1440 | 40  73 | 37  54 | 15  39 | 1.3 |
| 8^[b]^ | PLA | EtOH | 1.00 | 150 | 60 | 100 | 100 | 100 | – |
| 9^[b]^ | PLA | *n*-BuOH | 1.00 | 150 | 15  30 | 98  100 | 85  98 | 83  98 | –  – |
| 10^[b]^ | PLA | *i*-BuOH | 1.00 | 150 | 15  30 | 92  99 | 88  98 | 81  97 | –  – |
| 11^[b]^ | PLA | BnOH | 1.00 | 150 | 15  30  75 | 57  72  88 | 100  100  100 | 57  72  88 | –  –  – |
| 12^[b]^ | PLA | *t*-BuOH | 1.00 | 150 | 1440 | 29 | 15 | 4 | – |
| 13 | PCL | MeOH | 1.00 | 60  150^[b]^ | 1440  60 | 22  60 | 100  100 | 22  60 | –  – |
| 14 | PCL | EtOH | 1.00 | 60  150^[b]^ | 1440  60 | 24  80 | 100  100 | 24  80 | –  – |
| 15^[b]^ | PLA/  PCL blend | MeOH | [c] | 150 | 60 | 100(PLA)  18(PCL) | 100(PLA)  100(PCL) | 100(PLA)  18(PCL) | – |
| 16^[b]^ | PLA/  PCL blend | EtOH | [c] | 150 | 60 | 100(PLA)  14(PCL) | 100(PLA)  100(PCL) | 100(PLA)  14(PCL) | – |
| 17^[b]^ | PET | MeOH | 1.00 | 150 | 240 | – | – | 76^[f]^ | – |
| 18^[b]^ | PET | Ethylene glycol | 1.00 | 180 | 180 | – | – | 55^[f]^ | – |
| [a] Standard procedure: Schlenk tube, N_2_ atmosphere, 4 mL THF, 260 rpm, 1 mol % C1 (regarding the polymer ester bond), alcohol loading of 7 eq, 250 mg PLA (bio-mi Ltd.). [b] Solvent-free conditions. [c] 11.7 wt%. [d] Were calculated from ^1^H NMR spectroscopy according to literature. [e] Observable rate constant *k*_obs_ determined by plotting $\mathbf{ln}\left( \frac{\left[ \mathbf{LA} \right]_{\mathbf{0}}}{\left[ \mathbf{LA} \right]_{\boldsymbol{t}}} \right)$*vs*. t. [f] Isolated yield. | | | | | | | | | |

|  |  |
| --- | --- |

Figure S20: PLA methanolysis plot of conversion (PLA, Me-LA and CE (=chain end)) vs. time at 60 °C in THF using 1 mol% **C1** (left) and 2 mol% **C1** (right).

Figure S21: ^1^H NMR spectrum (CDCl_3_, 400 MHz) of the closed-loop recycling of PLA to LA using 1 mol% **C1** (regarding the polymer ester bonds) at 150 °C with vacuum after 24 h.

Figure S22: ^1^H NMR spectrum (CDCl_3_, 400 MHz) of the isolated lactide of the closed-loop recycling of PLA to LA using 1 mol% **C1** (regarding the polymer ester bonds) at 150 °C with vacuum after 24 h using a sublimation apparatus.

# **7. Computational results**

Table S12: Hirshfeld partial charges *δ*_H_ and steric parameters of selected atoms of the cation of **C1** and the cation of **CC2** as well as the zinc-coordinated lactone species (ORCA 6.0.0, TPSSh-D4/def2-TZVP/CPCM(THF)).

| Cationic species | *δ*_H_ (Zn) [e^-^] | *δ*_H_ (N_gua_) [e^-^] | *δ*_H_ (C_carbonyl; lactone_) [e^-^] | Buried Volume (Zn) [%] | Buried Volume (O_lactone_) [%] |
| --- | --- | --- | --- | --- | --- |
| **C1** | +0.355 | -0.209 | - | 79.51 | - |
| **C1**-LA | +0.368 | -0.211 | +0.221 | 80.45 | 57.23 |
| **C1**-CL | +0.401 | -0.204 | +0.227 | 80.66 | 55.62 |
| **CC2** | +0.346 | -0.211 | - | 78.33 | - |
| **CC2**-LA | +0.364 | -0.210 | +0.222 | 80.82 | 62.79 |
| **CC2**-CL | +0.358 | -0.216 | +0.225 | 80.69 | 61.68 |

Table S13: Hirshfeld partial charges *δ*_H_ for selected atoms of the zinc-coordinated lactide species of **C1, C2** and the cationic zinc-lactide complexes Zn-LA/Zn-CL (ORCA 6.0.0, TPSSh-D4/def2-TZVP/CPCM(THF)).

| Species | Charge | *δ*_H_ (Zn) [e^-^] | *δ*_H_ (C_carbonyl; lactone_) [e^-^] | *δ*_H_ (O_carbonyl; lactone_) [e^-^] | *δ*_H_ (N_gua_) [e^-^] |
| --- | --- | --- | --- | --- | --- |
| **C1**-LA | 2+ | +0.368 | +0.221 | -0.233 | -0.211 |
| **C2**-LA | 0 | +0.307 | +0.217 | -0.253 | -0.198 |
| Zn-LA | 2+ | +1.917 | +0.223 | -0.282 | - |


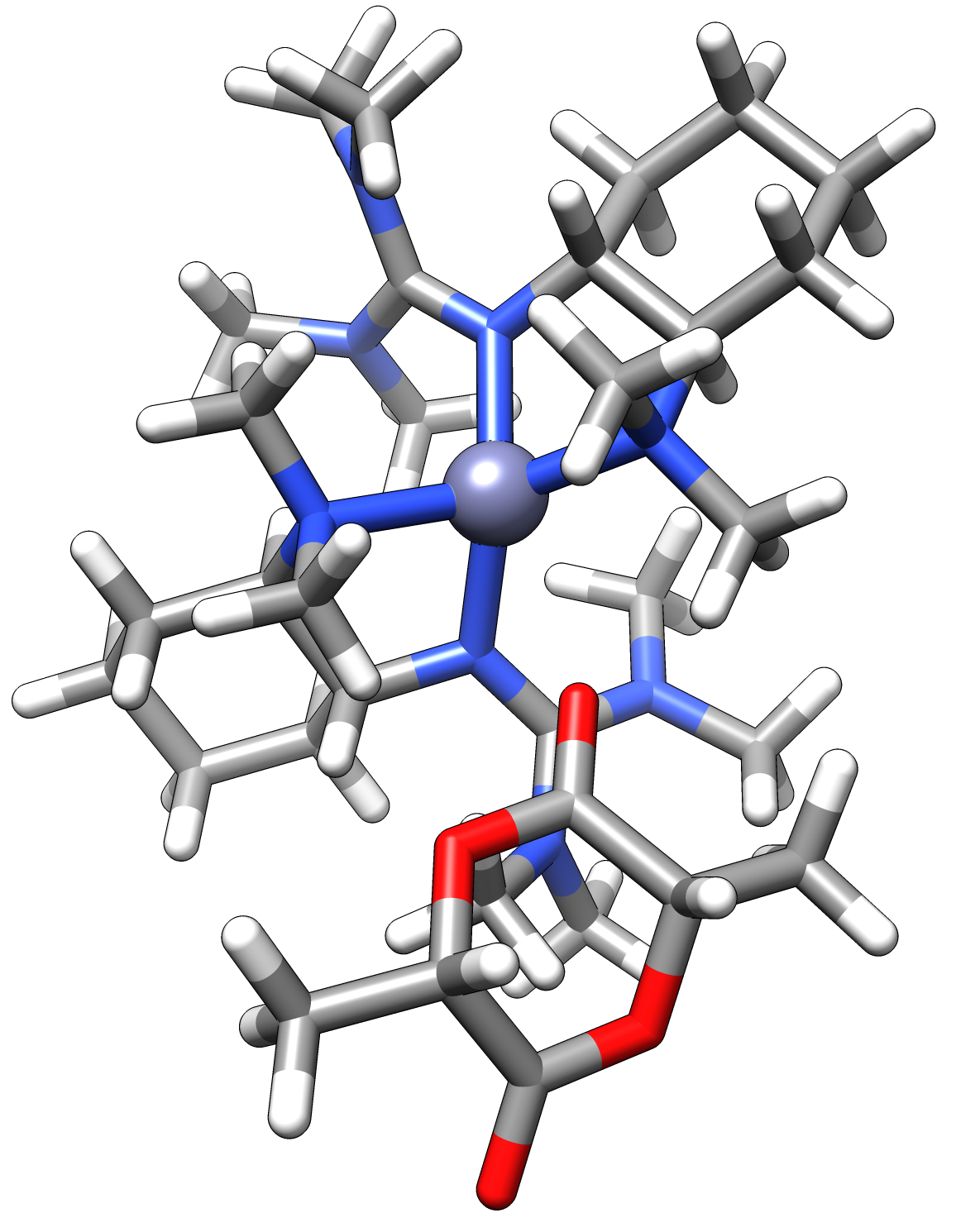


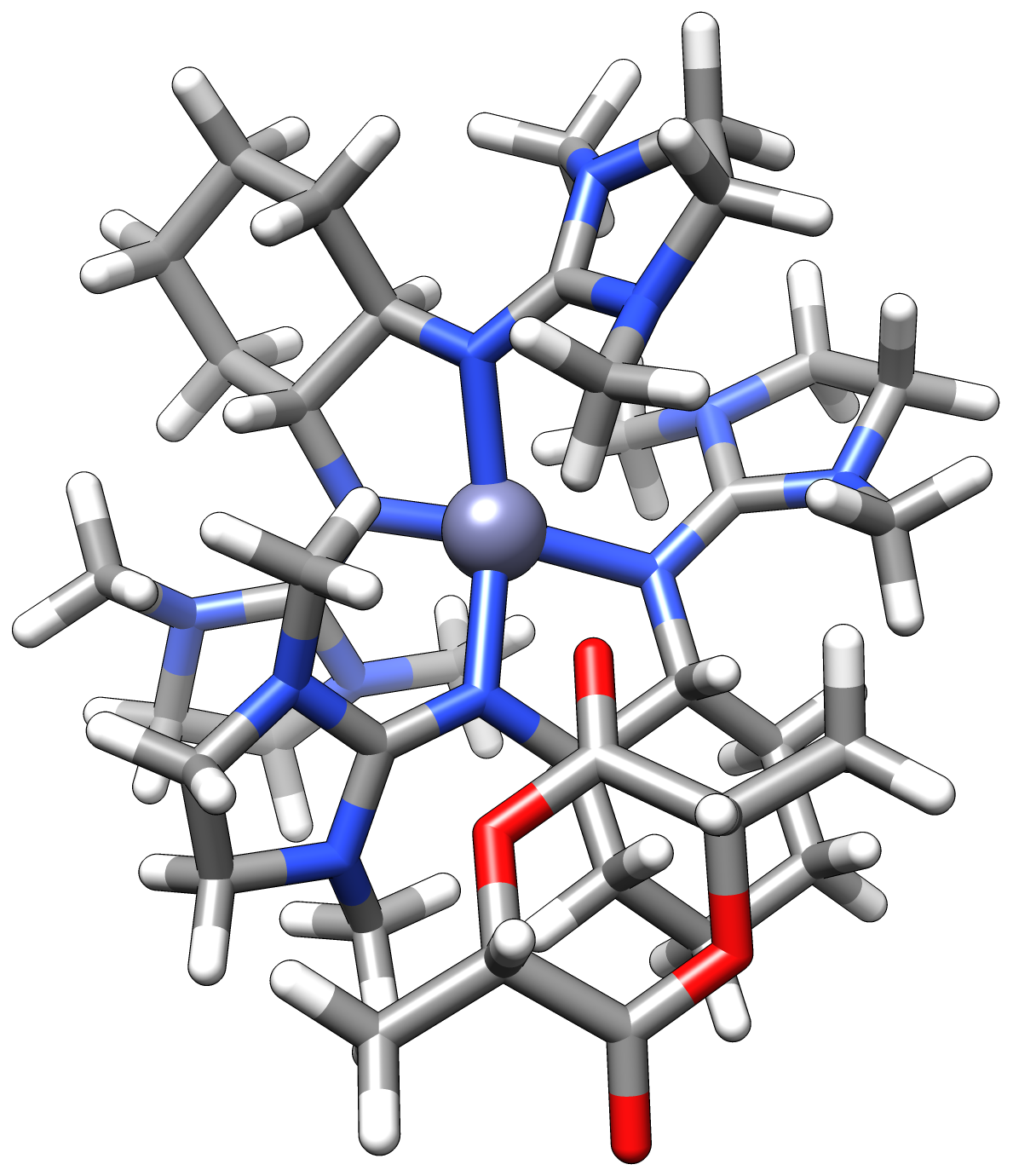


Figure S23: Computationally optimized cationic structures for the zinc-coordinated lactide for **C1** (left) and **CC2** (right).

# References

[1] K. Schwetlick, *Organikum*, Wiley-VCH Verlag GmbH, **2001**.

[2] H. Eilingsfeld, G. Neubauer, M. Seefelder, H. Weidincer, *Chem. Ber.* **1964**, *97*, 1232–1245.

[3] S. Herres-Pawlis, A. Neuba, O. Seewald, T. Seshadri, H. Egold, U. Flörke, G. Henkel, *Eur. J. Org. Chem.* **2005**, *2005*, 4879–4890.

[4] X-Area Pilatus3_SV 1.31.170.0, STOE, **2020**.

[5] X-Area Recipe 1.36.0.0, STOE, **2020**.

[6] X-Area Integrate 1.78.3.0, STOE, **2020**.

[7] X-Area LANA 1.83.8.0, STOE, **2020**.

[8] Bruker, XPREP, Bruker AXS Inc., Madison, Wisconsin, USA, **2007**.

[9] G. M. Sheldrick, Acta Crystallogr. *Sect. A* **1990**, 467–473.

[10] C. B. Hübschle, G. M. Sheldrick, B. Dittrich, *J. Appl. Crystallogr.* **2011**, *44*, 1281–1284.

[11] a) *SHELXT G M Sheldrick* **2015**, *Sect. A*, 3. b) *SHELXT G M Sheldrick* **2015**, *Sect. C*, 3.

[12] A. L. Spek, *A Multipurpose Crystallographic Tool, Utrecht University, Utrecht (The Netherlands)*, **2008**.

[13] A. L. Spek, Acta Crystallogr. **2009**, D65, 148–155.

[14] A. Kowalski, A. Duda, S. Penczek, *Macromolecules* **1998**, *31*, 2114–2122.

[15] M. Save, M. Schappacher, A. Soum, *Macromol. Chem. Phys.* **2002**, *203*, 889–899.

[16] S. Hardy, I. M. de Wispelaere, W. Leitner, M. A. Liauw, *Analyst* **2013**, *138*, 819–824.

[17] *NETZSCH, NETZSCH Proteus -Thermal Analysis (8.0.3), NETZSCH-Gerätebau GmbH, Selb, Germany*, **2022**.

[18] C. Bannwarth, S. Ehlert, S. Grimme, *J. Chem. Theory Comput.* **2019**, *15*, 1652–1671.

[19] C. Bannwarth, E. Caldeweyher, S. Ehlert, A. Hansen, P. Pracht, J. Seibert, S. Spicher, S. Grimme, *WIREs Comput. Mol. Sci.* **2021**, *11*, e1493.

[20] S. Ehlert, M. Stahn, S. Spicher, S. Grimme, *J. Chem. Theory Comput.* **2021**, *17*, 4250–4261.

[21] S. Grimme, C. Bannwarth, P. Shushkov, *J. Chem. Theory Comput.* **2017**, *13*, 1989–2009.

[22] M. Hymas, J. Eller, M. Salehi, R. Omidyan, S. Poigny, V. G. Stavros, *Phys. Chem. Chem. Phys.* **2025**, *27*, 15895–15905.

[23] S. Spicher, S. Grimme, *Angew. Chem. Int. Ed.* **2020**, *59*, 15665–15673.

[24] F. Neese, *WIREs Comput. Mol. Sci.* **2022**, *12*, e1606.

[25] V. N. Staroverov, G. E. Scuseria, J. Tao, J. P. Perdew, *J. Chem. Phys.* **2003**, *119*, 12129–12137.

[26] F. Weigend, R. Ahlrichs, *Phys. Chem. Chem. Phys.* **2005**, *7*, 3297–3305.

[27] E. Caldeweyher, C. Bannwarth, S. Grimme, *J. Chem. Phys.* **2017**, *147*, 034112.

[28] E. Caldeweyher, S. Ehlert, A. Hansen, H. Neugebauer, S. Spicher, C. Bannwarth, S. Grimme, *J. Chem. Phys.* **2019**, *150*, 154122.

[29] M. Cossi, B. Mennucci, J. Pitarch, J. Tomasi, *J. Comput. Chem.* **1998**, *19*, 833–846.

[30] A. M. Burow, M. Sierka, F. Mohamed, *J. Chem. Phys.* **2009**, *131*, 214101.

[31] G. L. Stoychev, A. A. Auer, R. Izsák, F. Neese, *J. Chem. Theory Comput.* **2018**, *14*, 619–637.

[32] F. Weigend, *Phys. Chem. Chem. Phys.* **2006**, *8*, 1057–1065.

[33] F. L. Hirshfeld, *Theor. Chim. Acta* **1977**, *44*, 129–138.

[34] J. P. Ritchie, *J. Am. Chem. Soc.* **1985**, *107*, 1829–1837.

[35] J. P. Ritchie, S. M. Bachrach, *J. Comput. Chem.* **1987**, *8*, 499–509.

[36] E. R. Davidson, S. Chakravorty, *Theor. Chim. Acta* **1992**, *83*, 319–330.

[37] P. Bultinck, C. Van Alsenoy, P. W. Ayers, R. Carbó-Dorca, *J. Chem. Phys.* **2007**, *126*, 144111.

[38] D. Geldof, A. Krishtal, F. Blockhuys, C. Van Alsenoy, *J. Chem. Theory Comput.* **2011**, *7*, 1328–1335.

[39] Luchini, T. Patterson, R. S. Paton, R. S. DBSTEP: DFT Based Steric Parameters **2022**. DOI: 10.5281/zenodo.4702097G

[40] A. K. Gupta, K. Raghavachari, *J. Phys. Chem. A* **2024**, *128*, 28–40.

[41] P. E. Bowling, S. Dasgupta, J. M. Herbert, *J. Chem. Inf. Model.* **2024**, *64*, 3912–3922.

[42] M. Bursch, J.-M. Mewes, A. Hansen, S. Grimme, **2022**, ChemRxiv preprint, DOI: 10.26434/chemrxiv-2022-n304h-v2.

[43] A. Hermann, T. Becker, M. A. Schäfer, A. Hoffmann, S. Herres-Pawlis, *ChemSusChem* **2022**, *15*, e202201075.

[44] A. Hermann, S. Hill, A. Metz, J. Heck, A. Hoffmann, L. Hartmann, S. Herres-Pawlis, *Angew. Chem. Int. Ed.* **2020**, *59*, 21778–21784; *Angew. Chem.* **2020**, *132*, 21962–21968.

[45] V. Raab, K. Harms, J. Sundermeyer, B. Kovačević, Z. B. Maksić, *J. Org. Chem.* **2003**, *68*, 8790–8797.

[46] L. Yang, D. R. Powell, R. P. Houser, *Dalton Trans.* **2007**, *0*, 955–964.

[47] M. H. Chisholm, S. S. Iyer, D. G. McCollum, M. Pagel, U. Werner-Zwanziger, *Macromolecules* **1999**, *32*, 963–973.

[48] T. M. McGuire, A. C. Deacy, A. Buchard, C. K. Williams, *J. Am. Chem. Soc.* **2022**, *144*, 18444–18449.

[49] J. M. Payne, M. Kamran, M. G. Davidson, M. D. Jones, *ChemSusChem* **2022**, *15*, e202200255.

[50] P. McKeown, L. A. Román-Ramírez, S. Bates, J. Wood, M. D. Jones, *ChemSusChem* **2019**, *12*, 5233–5238.

[51] L. A. Román-Ramírez, P. Mckeown, M. D. Jones, J. Wood, *ACS Catal.* **2019**, *9*, 409–416.

[52] L. Burkart, A. Eith, A. Hoffmann, S. Herres-Pawlis, *Chem. – Asian J.* **2023**, *18*, e202201195.
